# SARS-CoV-2 infection induces functional impairment of vitamin D receptor signaling to drive interleukin 6-dependent hyperinflammation in mononuclear phagocytes

**DOI:** 10.1371/journal.pone.0357030

**Published:** 2026-09-11

**Authors:** Juan Felipe Valdés-López, Diana di Filippo, Sharon Penagos, Lady Johana Hernández-Sarmiento, Johanna C. Arroyave-Ospina, Mauricio Rojas, Silvio Urcuqui-Inchima, Wbeimar Aguilar-Jiménez, Maria-Cristina Navas

**Affiliations:** 1 Grupo Gastrohepatología, Facultad de Medicina, Universidad de Antioquia, Medellín, Colombia; 2 Grupo Inmunovirología, Facultad de Medicina, Universidad de Antioquia, Medellín, Colombia; 3 Grupo de Inmunología Celular e Inmunogenética (GICIG), Facultad de Medicina y Unidad de Citometría, Sede de Investigación Universitaria, Universidad de Antioquia, Medellín, Colombia; Universitas Muhammadiyah Aceh, INDONESIA

## Abstract

The dysregulated inflammatory response, particularly the interleukin 6 (IL-6)-driven cytokine storm, is a hallmark of severe COVID-19. Mononuclear phagocytes are key cellular populations in the pathogenesis of SARS-CoV-2 infection, but the underlying regulatory mechanisms remain incompletely defined. Based on existing evidence of vitamin D immunomodulatory properties in viral infections, we investigated its role during SARS-CoV-2 infection. We integrated transcriptional profiling of monocytes and myeloid dendritic cells (mDCs) from healthy individuals and COVID-19 patients (moderate/severe, with/without viremia) with an *in vitro* model of SARS-CoV-2-infected monocyte-derived macrophages (MDMs). Additionally, a functional assay using U937-derived monocytes and macrophages challenged with inactivated SARS-CoV-2 particles was used to assess the immunomodulatory effect of calcitriol (vitamin D) treatment. We found that SARS-CoV-2 infection triggers an NF-κB-dependent inflammatory signature associated with disease severity, both in monocytes from COVID-19 patients and in MDMs infected *in vitro*. This signature is characterized by hyperproduction of IL-6 and upregulation of its signaling components, including IL6R, JAK1/2, STAT3, and SOCS3. Moreover, we found some evidence of a novel viral-dependent suppression of the Vitamin D Receptor (VDR) pathway, demonstrated by downregulation of both VDR expression and its target genes, including CAMP, LYZ, and IRF5, in monocytes from patients with COVID-19 and *in vitro* SARS-CoV-2-infected MDMs. This resulted in functional impairment of VDR signaling. Importantly, calcitriol treatment potently suppressed SARS-CoV-2-induced IL-6 production in our model of U937 cell line, suggesting that restoration of VDR signaling could temper this key inflammatory axis. Our findings reveal a dual-hit mechanism in severe COVID-19, in which SARS-CoV-2 infection simultaneously hyperactivates the pro-inflammatory pattern-recognition receptors/NF-κB/IL-6 axis and suppresses the anti-inflammatory VDR pathway in mononuclear phagocytes. The effective inhibition of IL-6 by calcitriol provides a potential role of vitamin D in mitigating pathological inflammation, positioning it as a plausible immunomodulatory strategy for severe COVID-19.

## Introduction

Coronavirus Disease 2019 (COVID-19) is an infectious disease caused by Severe Acute Respiratory Syndrome Coronavirus 2 (SARS-CoV-2), a single-stranded positive-sense RNA virus belonging to the *Coronaviridae* family [1]. While most COVID-19 cases are asymptomatic or cause mild respiratory symptoms, a significant subset of patients, approximately 20%, develop severe pneumonia, often progressing to acute respiratory distress syndrome (ARDS), systemic hyperinflammation, and multi-organ failure [2,3].

A hallmark of severe COVID-19 is the dysregulated and systemic release of pro‑inflammatory cytokines, a phenomenon described as “cytokine storm”. In fact, mortality in COVID-19 is largely driven by immunopathogenesis mechanisms rather than by viral cytopathic effects [2,4]. This hyperinflammatory state disrupts endothelial integrity, increases vascular permeability, promotes the activation and migration of immune cells, and accumulates protein‑rich fluid in alveolar spaces, thereby driving and worsening clinical outcomes [2,4,5]. Moreover, SARS‑CoV‑2 infection triggers robust NF‑κB‑dependent transcription of inflammatory mediators, including interleukin-1β (IL‑1β), IL‑6, IL‑8/CXCL‑8, and tumoral necrosis factor-alpha (TNF‑α) [6–8]. Notably, high serum levels of IL‑6 and IL‑8/CXCL‑8 strongly correlate with severity and poor prognosis, suggesting that these cytokines could serve as prognostic biomarkers [9–13]. Nevertheless, the mechanisms of immunopathogenesis linking innate immune dysregulation to the highly variable clinical spectrum of COVID‑19 remain incompletely understood.

Mononuclear phagocytes, including monocytes, macrophages, and myeloid dendritic cells (mDCs), are central players in COVID‑19 pathogenesis [8,14–16]. These cells are direct and indirect targets of SARS‑CoV‑2 that express a broad repertoire of pattern‑recognition receptors (PRRs). Key PRRs include Toll-like receptors (TLRs) such as TLR2 and TLR4, which sense the SARS-CoV-2 Envelope (E) and Spike (S) glycoproteins [6,14,17], and TLR3, which recognizes double-stranded RNA (dsRNA) replication intermediates [18]. Other important families are NOD-like receptors (NLRs), activated by viral proteins [19–21], and cytosolic RNA sensors such as retinoic acid-inducible gene I (RIG-I) and melanoma differentiation-associated protein 5 (MDA5) [22]. While PRR engagement initiates antiviral responses, hyperactivation in severe COVID‑19 drives excessive inflammation and cytokine storm [4,5,12]. Thus, fine‑tuning mononuclear phagocyte activity represents a promising therapeutic strategy to mitigate immunopathology.

Vitamin D (VD) is an immunomodulator with broad effects on innate and adaptive immunity [23–26]. Its biological activity depends on a tightly regulated two-step enzymatic activation where the precursor cholecalciferol is hydroxylated by 25-hydroxylases (CYP2R1, CYP27A1) to form 25(OH)D3 (calcidiol) in the liver, and subsequently by 1α-hydroxylase (CYP27B1) in the kidneys to produce the active hormone 1α,25(OH)_2_D3 (calcitriol) [27–29]. Beyond its classical role in calcium homeostasis, VD signals through the nuclear vitamin D receptor (VDR), which heterodimerizes with Retinoid X receptor alpha (RXRα) to regulate gene transcription [30–32]. In mononuclear phagocytes, VD enhances antimicrobial defenses by inducing the antimicrobial peptide cathelicidin (CAMP/LL-37) [33,34], while suppressing pro‑inflammatory cytokine production, including TNF‑α, IL‑6, and IL‑8/CXCL-8 by inhibiting NF‑κB signaling [26,31,35–37]. These cells possess enzymatic machinery for VD activation (CYP27B1), supporting an autocrine/paracrine immunoregulatory loop [38]. Low VD levels are associated with increased frequency of respiratory infections and worse COVID‑19 outcomes, suggesting a protective role [39]. Notably, Calcitriol has been shown *in vitro* to inhibit viral replication of human immunodeficiency virus‑1 [40,41], dengue virus [34,37,42], and respiratory syncytial virus [43]. In addition, VD attenuates inflammatory responses in monocytes and macrophages infected with dengue [34,37,42], chikungunya [35], and zika viruses [44,45]. Furthermore, low circulating VD levels have been associated with elevated levels of TNF‑α, IL-6, and IFN-γ in dengue patients with warning signs and severe dengue [46]. However, its specific impact on SARS‑CoV‑2‑driven inflammation and the underlying molecular mechanisms, particularly in primary human mononuclear phagocytes, remain poorly defined.

Given the central role of mononuclear phagocytes in COVID‑19 pathogenesis and the anti‑inflammatory properties of vitamin D, we hypothesized that SARS‑CoV‑2 dysregulates VD–VDR signaling in these cells, thereby exacerbating NF‑κB‑dependent inflammation. To test this, we integrated transcriptomic data from *ex vivo* analyses of monocytes and myeloid dendritic cells (mDCs) obtained from COVID‑19 patients, with *in vitro* models of monocyte‑derived macrophages (MDMs) infected with SARS‑CoV‑2 or stimulated with virus-like particles (VLPs). We characterized PRR expression, NF‑κB pathway activation, and inflammatory cytokine production, and evaluated VD‑VDR pathway activity in different stages of disease severity. Furthermore, we performed *in vitro* functional assays using U937-derived monocytes and macrophages treated with calcitriol and challenged with ultraviolet (UV)-inactivated SARS-CoV-2 particles to determine whether vitamin D modulates SARS‑CoV‑2‑induced cytokine production.

## Materials and methods

### Ethics statement

This study was designed and conducted in accordance with the Declaration of Helsinki and Colombian legislation (Colombian Ministerio de Salud Resolution 008430 of 1993).

This study is a re-analysis of publicly available data, and that the original studies obtained proper ethical approval. No new human samples were collected by the authors. Further, this study received institutional endorsement and followed biosafety protocols for inactivated viral particles and cell culture handling. The authors confirm that all methods were carried out in accordance with relevant guidelines and regulations.

### Bioinformatic analysis of previously published mRNA datasets and prediction of cell signaling pathway activation

To investigate the transcriptional profiling of mononuclear phagocytes in response to SARS-CoV-2 infection, and the transcriptional profile of U937-derived macrophages, we reanalyzed three publicly available RNA-Seq datasets. The first (GSE216529), available in the GEO (https://www.ncbi.nlm.nih.gov/gds/?term=GSE216529) and published by Sun et al. [47], comprises RNA-seq data from patients with moderate COVID-19 without detectable viremia, severe COVID-19 with or without detectable viremia, and healthy controls, focusing only on monocytes and mDC, with n = 4 per group. The second dataset (GSE224845), available in the GEO (https://www.ncbi.nlm.nih.gov/gds/?term=GSE224845) and generated by Simón-Fuentes et al. [48], includes RNA-seq data from human GM-CSF-dependent MDMs exposed to SARS-CoV-2 (clinical isolate corresponding to the ancestral S D614G variant, GISAID accession EPI_ISL_1120962; MOI = 1) or to equivalent amounts of SARS-CoV-2-derived virus-like particles (VLPs) for 4, 12, and 36 hours (n = 3 or 4). The third dataset GSE308204, available in the GEO (https://www.ncbi.nlm.nih.gov/gds/?term=GSE308204) and generated by Panahipour et al. [49], This dataset was generated from U937 and THP-1 cells differentiated into macrophages with 10 ng/mL phorbol-12-myristate-13-acetate (PMA) for 48 hours. The metadata for the different transcriptomes are summarized in Table 1.

**Table 1:** Metadata of bulk RNA-seq employed in the study.

GEO accession	Sample type	Experimental groups (n)	Disease model	Sequencing technology	Experimental condition	Patient metadata available
**GSE216529**	FACS-sorted peripheral blood immune cell populations (CD4 T, CD8 T, B, NK, monocytes, mDC, pDC), from PBMCs	Moderate COVID-19 without viremia (n = 4) Severe COVID-19 without viremia (n = 4) Severe COVID-19 with plasma viremia (n = 4) Healthy donors (n = 4)	COVID-19	Bulk RNA-seq NextSeq 550 (Homo sapiens) Sequences were mapped to GRCh38 genome	Comparison of immune-cell transcriptomes according to COVID-19 severity and plasma viremia status	Disease severity, plasma viremia status, immune cell type, patient ID. No detailed demographics (age, sex, comorbidities) are deposited in the GEO Series metadata.
**GSE224845**	Human GM-CSF-derived macrophages (in vitro differentiated)	Mock control (n = 4 per time point) SARS-CoV-2 infection (n = 4 per time point) SARS-CoV-2 virus-like particles (VLPs) (n = 4 per time point) Time points (4, 12, and 36 h.p.i.)	SARS-CoV-2 infection/VLP stimulation	Bulk RNA-seq BGISEQ-500 (Homo sapiens) Sequences were mapped to GRCh38 genome	Transcriptional effects induced by infectious SARS-CoV-2 and VLPs in human macrophages	Donor identifiers and experimental condition are available. Limited donor characteristics are deposited; no comprehensive clinical metadata reported.
**GSE308204**	PMA-differentiated U937 macrophages and THP-1 macrophages	U937 macrophages, MOCK condition (n = 3) PRF lysate-treated (n = 3) THP-1 macrophages, MOCK condition (n = 3) PRF lysate-treated (n = 3)	PRF-induced inflammatory polarization	Bulk RNA-seq Illumina HiSeq 2000 (Homo sapiens) Sequences were mapped to GRCh38 genome	Platelet-rich fibrin (PRF) exposure to investigate inflammatory macrophage polarization	Not applicable (cell-line study). No patient metadata because samples derive from immortalized cell lines.

To determine the differentially expressed genes (DEG), we selected genes with adjusted p-value < 0.05, and |Log2 Fold Change (SARS-CoV-2-infected cells/unstimulated cells) | > 1.0 (|log2FC| > 1.0), using the DESeq2 library [50]. Then, the raw counts were normalized to transcript per million (TPM) using R statistical software (version 4.2.0). Genes associated with the immunophenotype of mononuclear phagocytes, PRRs signaling, NF-κB complex, NF-κB target genes, and the VD-VDR signaling pathway were selected as previously described [35,51], and the data were visualized using box plots and heatmaps generated with R statistical software (version 4.2.0) and GraphPad Prism for Windows (GraphPad Software version 8.0.1, California, USA; www.graphpad.com).

### SARS-CoV-2 stock, viral titration, and UV-light inactivation

A clinical isolate of SARS-CoV-2 obtained from a Colombian patient [ancestral B.1 lineage (EPI_ISL_536399)] was *in vitro* replicated at the SIU-University of Antioquia biosafety level 3 (BSL-3). The viral stocks were produced in a monolayer of Vero-E6 cells (ATCC), maintained in Dulbecco’s Modified Eagle Medium (DMEM; Sigma-Aldrich) supplemented with 5% heath-inactivated fetal bovine serum (FBS; Sigma-Aldrich), and 1% (v/v) penicillin/streptomycin solution (Sigma-Aldrich) at 37°C in a 5% CO_2_ atmosphere. After an advanced cytopathic effect was detected, viruses were collected, cleared of cell debris by centrifugation (2500 rpm/10 min), and stored at −80°C. Viral titers were determined by plaque-forming assay on Vero-E6 cells, as previously described [52]. SARS-CoV-2 was inactivated by UV radiation: 200 µL of virus were exposed to UV light at 10 cm (~365 nm, ~ 145 mW/cm^2^) for 120 min. The UV-inactivated virus was stored at −80°C, and complete inactivation was confirmed by plaque assay on Vero E6 cells.

### U937 cell culture, SARS-CoV-2 challenged, and vitamin D stimulation

The human monocytic U937 cell line (ATCC) was maintained at a density of 1 × 10^5^–1 × 10^6^ cells/mL in RPMI-1640 medium (Sigma-Aldrich) supplemented with 5% fetal bovine serum (FBS) and 1% (v/v) penicillin/streptomycin at 37°C in a 5% CO_2_ atmosphere in cell culture flasks. For cell differentiation, U937 cells (5 × 10^5^ cells/well) were seeded in 24-well plates and treated with or without 100 nM phorbol 12-myristate13-acetate (PMA; Sigma-Aldrich) for 3 days at 37°C in a 5% CO_2_ atmosphere to obtain U937-derived macrophages and U937-derived monocytes, respectively. Cell morphotypes were visualized and photographed in an Axio Vert.A1 microscope (ZEISS, Germany), adapted to transmitted light in phase-contrast microscopy. To confirm monocyte/macrophage immunophenotype, U937 cells were incubated for 40 min at 4°C in presence of monoclonal mouse anti-human CD14-Fluorescein isothiocyanate (FITC;2ng/μL; clone: 61D3), CD11b/Mac1-PE/Cyanine5 (PE/Cy5; 2ng/μL; clone: ICRF44), or the relevant isotype antibodies (eBioscience, USA). A BD LSRFortessa flow cytometer (BD Biosciences, USA) was used to obtain reading of the experiment, and the FlowJo-v10 software was used to analyze the data.

Then, cells were challenged with UV-inactivated SARS-CoV-2 particles (MOI 0.5) and treated with or without 10 nM Calcitriol (VD; Sigma-Aldrich) during (0h), 2 hours before (-2h), or 2 hours after (+2 h) SARS-CoV-2 challenge. Cells stimulated with 100 ng/mL ultrapure *E. coli* lipopolysaccharide (LPS, a TLR4 agonist; Sigma-Aldrich) served as a positive control. Culture supernatants were collected at 4- or 24-hours post-stimulation (hps) and stored at −80°C.

### Cytokine quantification

The Human Inflammatory Cytokines Cytometric Bead Array (CBA) Kit (BD Biosciences) was used for the detection of IL-1β, IL-6, IL-8/CXCL-8, IL-10, IL-12p70, and TNF-α in culture supernatants of U937-derived monocytes and macrophages treated or not with calcitriol, and stimulated with UV-inactivated SARS-CoV-2 particles, following the manufacturer’s instructions. The detection limit was 20 pg/mL. A BD LSRFortessa flow cytometer was used to obtain the reading of the experiment. A cytokine kinetics assay was previously carried out to select the collection times (4, 6, 12 or 24 hps, data not shown).

### Statistical analysis

Statistical analysis was performed using GraphPad Prism for Windows (GraphPad Software version 8.0.1, San Diego, CA, USA; www.graphpad.com). The Shapiro-Wilks test was performed to determine the normality of the data. Data are represented as mean ± SEM or as median with interquartile range. The statistical tests are indicated in the fig legends. Significant results were for the Kruskal-Wallis test with multiple comparisons, *p* < 0.05 (*).

## Results

### Monocytes and mDCs display a severity-associated immunophenotypic dysregulation during COVID-19

**Fig 1:**
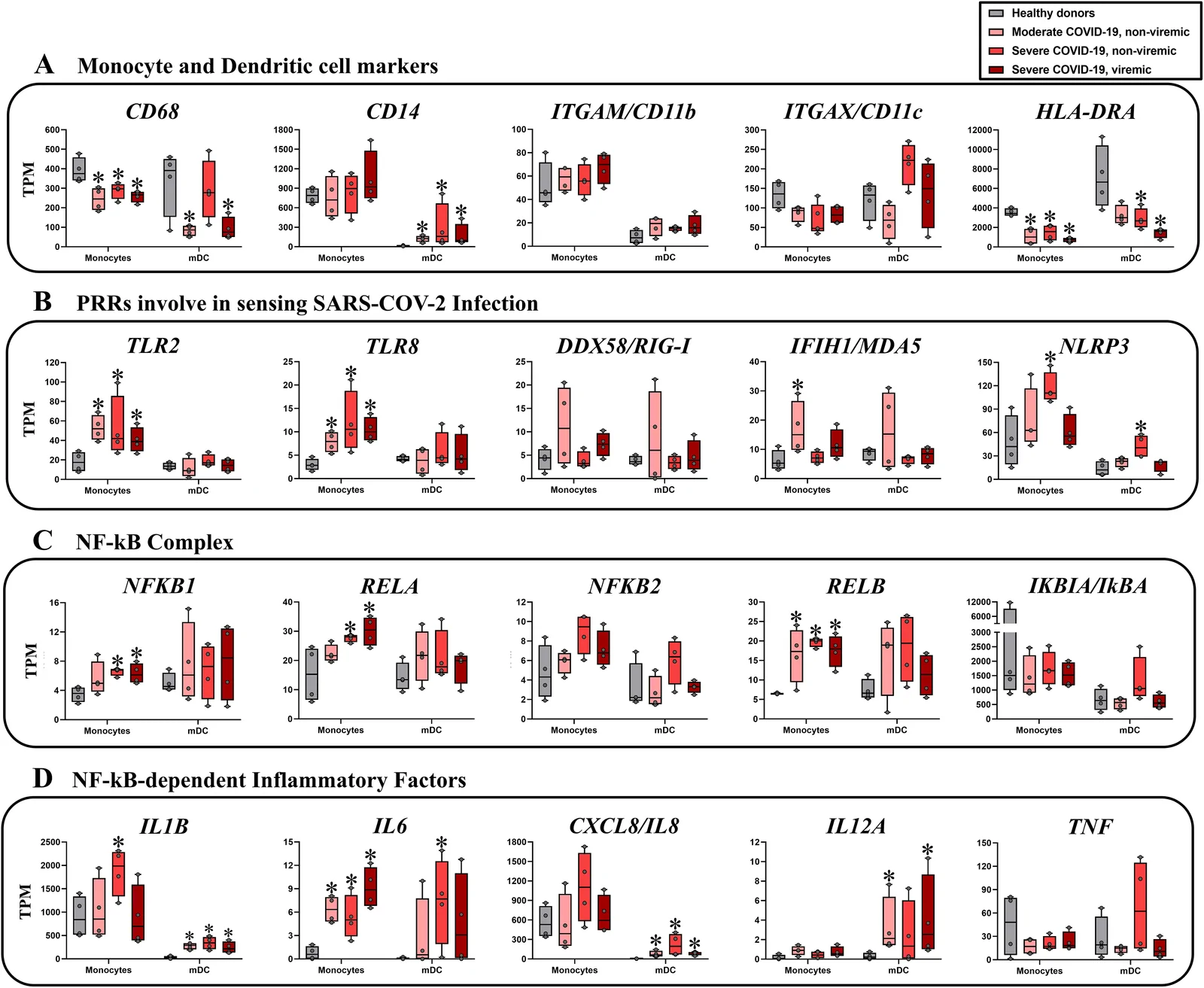
Expression of myeloid and inflammatory markers in human monocytes and mDCs is associated with COVID-19 severity. We reanalyzed the publicly available RNA-Seq dataset GSE216529 (GEO); see Methods o Table 1. Gene expression was quantified as Transcripts Per Million (TPM). Depicted is the mRNA abundance of (A) monocyte and dendritic cell markers, (B) pattern recognition receptors (PRRs) involved in SARS-CoV-2 sensing, (C) the NF-κB complex, and (D) canonical NF-κB target genes. Data is presented as medians with interquartile range. Normality was assessed using the Shapiro-Wilk test, and group comparisons were performed using the Kruskal-Wallis test followed by Dunn's post-hoc test (uncorrected). Significant differences (p < 0.05) between healthy individuals and patient groups are indicated by an asterisk (*).

Previous studies have shown that mononuclear phagocytes, particularly monocytes and macrophages, contribute directly to both the immune response and pathogenesis of SARS-CoV-2 infection [8,14–16]. We initially analyzed transcriptional profiles in monocytes and mDCs from healthy individuals and patients with moderate and severe COVID-19, with or without detectable viremia (n = 4 donors per group), using the publicly available dataset GSE216529 [47]. We first confirmed the immunophenotype of both cell populations by assessing transcriptional expression of *CD68*, *CD14, ITGAM/CD11b*, *ITGAX/CD11c*, and *HLA-DRA* (Fig 1A). SARS-CoV-2 infection was associated with significant modulation of these markers in patients; indeed, a significant decrease in *CD68* mRNA expression was observed in mDCs from patients with severe COVID-19 with viremia and from patients with moderate COVID-19 without viremia. *CD68* expression was also downregulated in monocytes from COVID-19 patients, regardless of disease severity. Additionally, increased *CD14* mRNA expression was observed in mDCs from infected patients, but not in monocytes (Fig 1A). No significant changes were observed in *ITGAM/CD11b* or *ITGAX/CD11c* mRNA levels in either monocytes or mDCs. While *HLA-DRA* mRNA expression was downregulated in monocytes from all COVID-19 patients and in mDCs from patients with severe diseases. Collectively, these results suggest that both monocytes and mDCs exhibit a distinct state of immune activation in COVID-19 patients, depending on disease severity.

### Distinct PRR signaling and NF-κB-driven inflammatory responses in monocytes and mDCs are associated with COVID-19 severity

We next evaluated the modulation of PRRs expression in monocytes and mDCs from COVID-19 patients. We observed a significant upregulation of *TLR2* and *TLR8* in monocytes from COVID-19 patients, regardless of disease severity, whereas their expression did not change in mDCs (Fig 1B). We also identified significantly increased *NLRP3* mRNA expression on monocytes and mDCs from patients with severe COVID-19 without viremia (Fig 1B). In contrast, neither monocytes nor mDCs from severe COVID-19 patients showed significant modulation in *DDX58/RIG-I* or *IFIH1/MDA5 mRNA* expression (Fig 1B). Notably, monocytes from patients with moderate COVID-19 exhibited significantly higher *IFIH1/MDA5* mRNA expression compared with those from patients with severe COVID-19 or healthy donors. Consistent with these PRR findings, we observed a significant, severity-dependent increase in the expression of canonical NF-κB signaling components in monocytes (Fig 1C). *NFKB1* and *RELA* mRNA expression was significantly increased in monocytes from patients with severe COVID-19, regardless of viremia status, whereas their expression did not change in mDCs, independent of disease severity. *RELB* mRNA expression was significantly higher in monocytes from COVID-19 patients, independent of severity (Fig 1C). Non-significant changes were observed in *IKBIA/IKBA* mRNA expression in both populations*.* In line with this result, *IL1B* mRNA expression was significantly induced in monocytes from patients with severe COVID-19 without viremia (Fig 1D). Furthermore, *IL6* mRNA expression was upregulated in monocytes from COVID-19 patients regardless of disease severity (Fig 1D). These findings confirm that monocyte activation during SARS-CoV-2 infection promotes NF-κB signaling and the induction of inflammatory mediators. In mDCs, the expression of classical NF-κB target genes, including *IL1B* and *CXCL8/IL8*, was also increased, although these changes were not associated with disease severity (Fig 1D). In addition, *IL12A* mRNA expression was significantly increased in mDCs from patients with moderate COVID-19 without viremia and those with severe COVID-19 with viremia (Fig 1D). Finally, *TNF* mRNA expression shows no changes in either cell population, regardless of disease severity. These results indicate that SARS-CoV-2 infection drives systemic activation of monocytes and, to a lesser extent, mDCs, which actively contribute to the inflammatory response through NF-κB-dependent mechanisms.

### Monocytes, but not mDCs, exhibit a severity-associated autocrine IL-6 signaling signature in COVID-19

**Fig 2:**
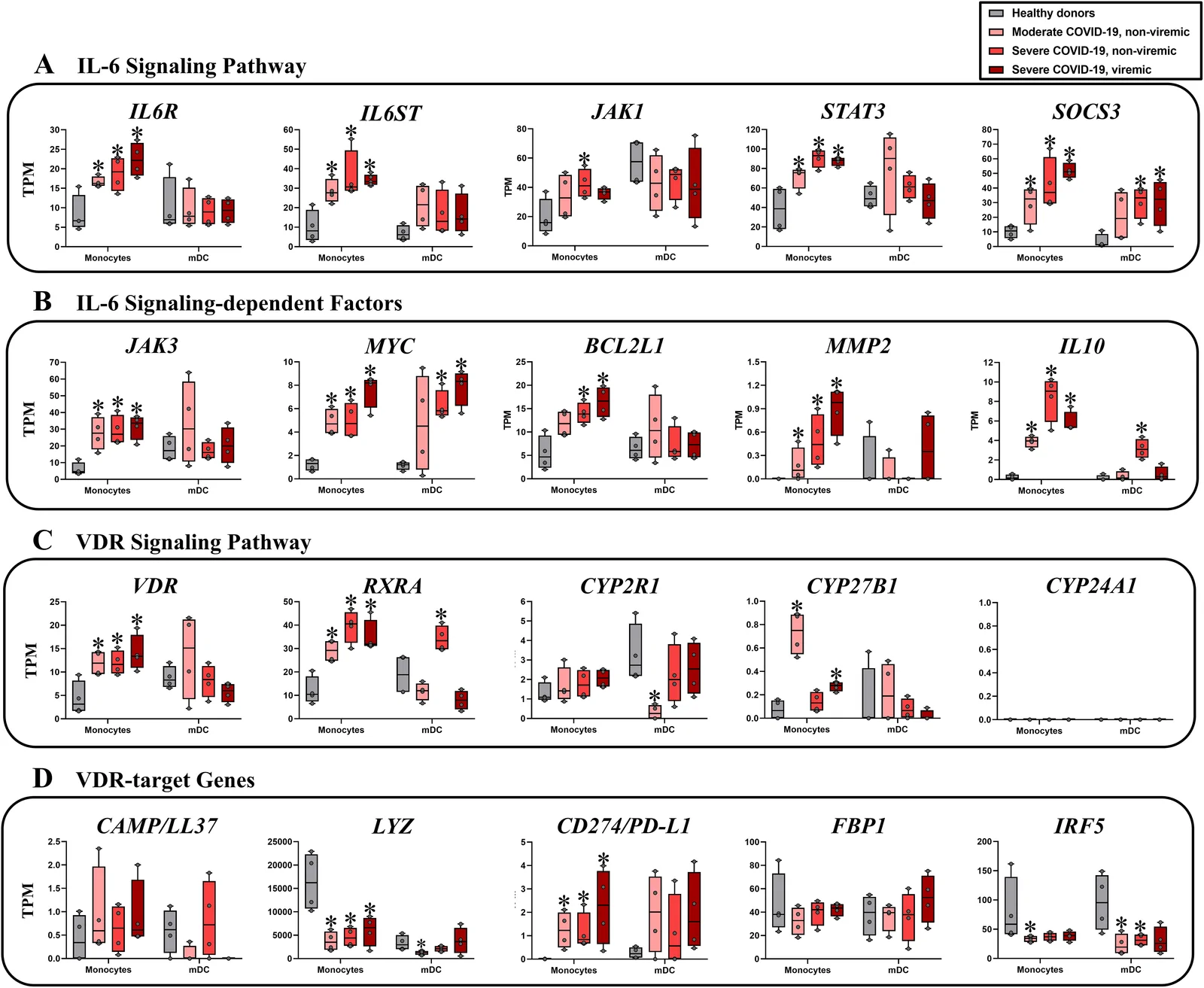
Expression of IL-6- and VDR-dependent signaling components in human monocytes and mDCs is associated with COVID-19 severity. We reanalyzed the publicly available RNA-Seq dataset GSE216529 (GEO); see Methods or Table 1. Gene expression was quantified as Transcripts Per Million (TPM). Shown is the mRNA abundance of (A) core IL-6 signaling components, (B) IL-6-induced inflammatory factors, (C) the VDR signaling pathway, and (D) canonical VDR-target genes. Data is presented as medians with interquartile range. After assessing normality with the Shapiro-Wilk test, group comparisons were performed using the Kruskal-Wallis test with Dunn’s post-hoc test (uncorrected). Significant differences (p < 0.05) between healthy individuals and patient groups are indicated by an asterisk (*).

Although IL-6 has been previously linked to SARS-CoV-2 infection severity and serum cytokine levels have been correlated with disease progression [4,11,12,47], the functional role of this cytokine in driving the inflammatory response in COVID-19 patients remains incompletely elucidated. Based on *IL6* mRNA expression (Fig 1D), we found increased mRNA expression of IL-6 signaling components in monocytes from COVID-19 patients, including the IL-6 receptor subunits (*IL6R*, *IL6ST*), the transcription factor *STAT3*, and the negative regulator of the pathway, *SOCS3*, in line with the severity of the disease (Fig 2A). Consistent with these findings, we observed a significant upregulation of canonical IL-6 signaling target genes [53,54], in monocytes from COVID-19 patients, including Janus kinase 3 (*JAK3*), MYC oncogene (*MYC)*, BCL-2-like protein 1 (*BCL2L1)*, Matrix metalloproteinase-2 (*MMP2)*, and *IL10* (Fig 2B). In contrast, mDCs showed a significantly upregulated of *SOCS3* mRNA expression only in patients with severe COVID-19. This finding suggests that monocytes, but not mDCs, from COVID-19 patients robustly activate the IL-6 signaling pathway.

### SARS-CoV-2 infection dysregulated Vitamin D Receptor signaling in monocytes, potentially contributing to inflammatory response in patients

Previous reports have shown that low serum VD levels correlate with worse outcomes in respiratory viral infections, including COVID-19 [39,55]. However, the effect of SARS-CoV-2 infection on the expression of components involved in VD biosynthesis and signaling remains uncharacterized. Here, we identified a significant increase in *VDR* and *RXRA* mRNA expression in monocytes from COVID-19 patients, and expression of *CYP27B1*, a key enzyme in the VD biosynthesis pathway, was increased in monocytes from patients with moderate and severe COVID-19 with viremia (Fig 2C). In contrast, no significant changes were observed in *VDR mRNA* expression or enzymes involved in VD biosynthesis or catabolism in mDCs, except for *RXRA*, which was upregulated in patients with severe COVID-19 without viremia, and *CYP2R1*, which was downregulated in patients with moderate COVID-19 (Fig 2C). The enzyme involved in vitamin D catabolism, *CYP24A1*, was not significantly induced in monocytes and mDCs from healthy individuals or patients with COVID-19 (Fig 2C). This finding may suggest that VD signaling plays a more relevant role in modulating monocyte responses than mDCs responses. Although we identified increased expression of both VDR subunits in monocytes from COVID-19 patients, we observed a significant downregulation of canonical VDR target genes expression [35,56], including Lysozyme (*LYZ*), Interferon-regulatory factor 5 (*IRF5)* (Fig 2D), and *CD14* (Fig 1A). This finding was accompanied by minimal induction of the antimicrobial peptide *CAMP/LL37* and the metabolic effector fructose-1,6-bisphosphatase 1 (*FBP1),* but a high and significant induction of Programmed death-ligand 1 *(CD274/PD-L1)* (Fig 2D). In mDCs, the expression of *IRF5* was significantly downregulated in moderate and severe COVID-19 patients without viremia, whereas *LYZ* mRNA was downregulated only in patients with moderate COVID-19 (Fig 2D). Together, these results suggest that SARS-CoV-2 infection could dysregulated VDR signaling in human monocytes, an effect that may be linked to the severity of the inflammatory response in COVID-19 patients.

### SARS-CoV-2 infection drives NF-κB-mediated inflammation in human macrophages while suppressing antigen presentation

**Fig 3:**
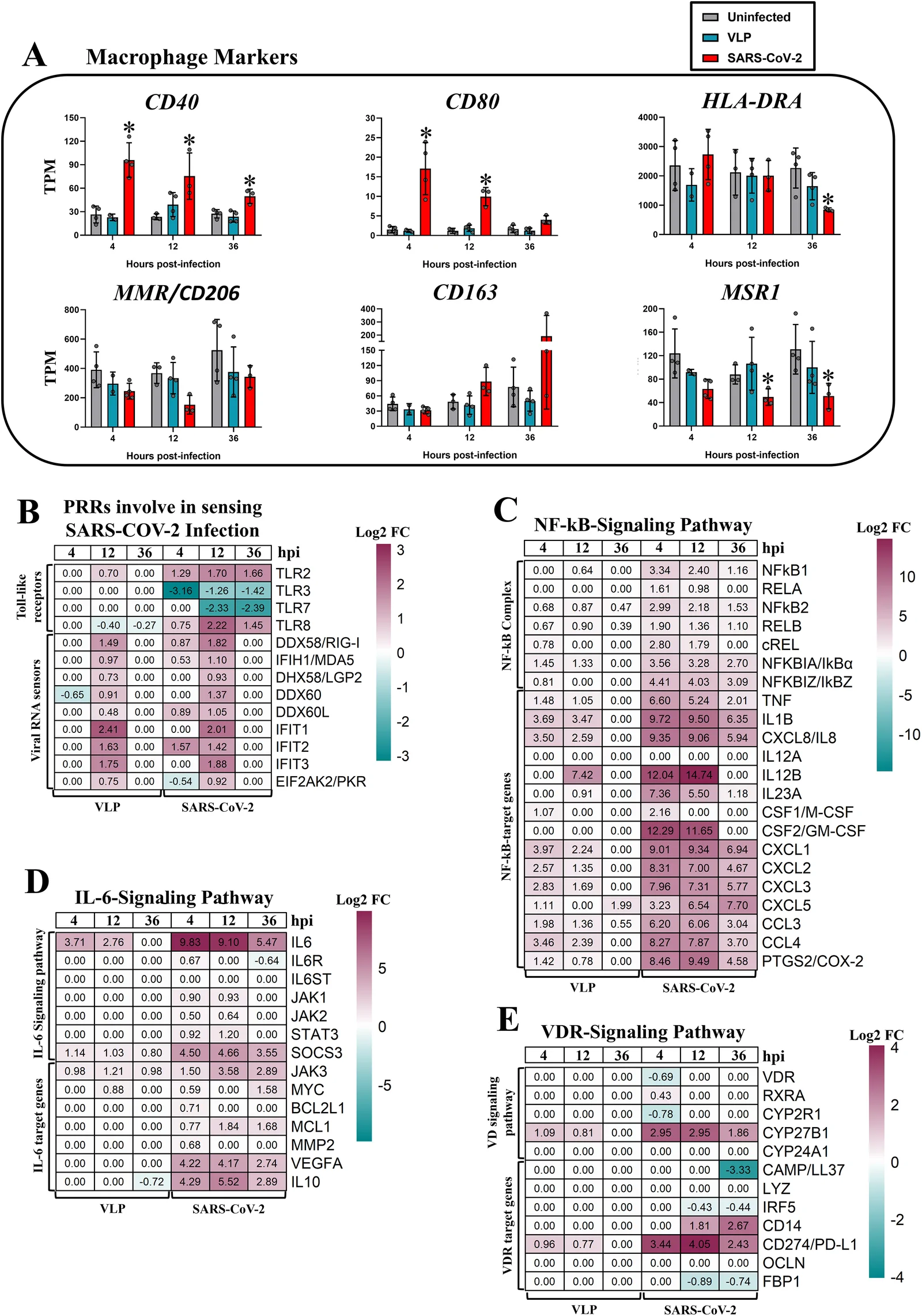
SARS-CoV-2 infection promotes a pro-inflammatory profile and a robust NF-κB-dependent inflammatory response in human macrophages. We reanalyzed the publicly available RNA-Seq dataset GSE224845 (GEO); see Methods o Table 1. Gene expression is shown as Transcripts Per Million (TPM) and in Log2FC heatmaps. Shown are the mRNA expression levels of (A) Macrophages markers, (B) pattern recognition receptors (PRRs) involved in SARS-CoV-2 sensing, (C) the NF-κB signaling pathway, (D) the IL-6 signaling pathway, and (E) the VDR signaling pathway. TPM data are presented as mean ± SD. Normality was assessed using the Shapiro-Wilk test, and statistical comparisons were performed using the Kruskal-Wallis test followed by Dunn's post-hoc test (uncorrected). Significant differences (p < 0.05) between MOCK and SARS-CoV-2-exposed cells are indicated by an asterisk (*).

Macrophages have also been proposed as key players in COVID-19 immunopathogenesis [8,14–16]. Indeed, we previously demonstrated that stimulation of MDMs with recombinant SARS-CoV-2 Spike protein induces a robust inflammatory response dependent on NF-κB activation [8]. Taking into account that the transcriptional response of MDMs to SARS-CoV-2 infection remains incompletely understood, we reanalyzed the publicly available GSE224845 dataset [48], generated from GM-CSF-differentiated MDMs infected with SARS-CoV-2 (MOI = 1), or stimulated with an equivalent amount of SARS-CoV-2-derived VLPs, for 4, 12, or 36 hours. In agreement with previous reports [57–59], the transcriptomic analysis showed high expression level of classical inflammatory macrophage markers, including co-stimulatory molecules such as *CD40* and *CD80* (Fig 3A). Their expression increased in response to SARS-CoV-2 infection but not to VLPs, with a peak in mRNA expression at 4 hours post-infection (hpi; Fig 3A). Although *HLA-DRA* mRNA expression was unmodified in both MDMs infected with SARS-CoV-2 or cultivated with VLPs (Fig 3A), its expression was significantly reduced following SARS-CoV-2 infection at 36 hpi (Fig 3A). This phenotype mirrors that observed in monocytes and mDCs from COVID-19 patients (Fig 1A), consistent with downregulation of *HLA-DRA* expression in SARS-CoV-2 infection in mononuclear phagocytes both *in vitro* and *in vivo*. Furthermore, in agreement with previous reports describing the expression of phagocytic receptors, including the macrophage mannose receptor (*MRC1/CD206*), *CD163*, and Macrophage scavenger receptor 1 (*MSR1*) [57–59], neither SARS-CoV-2 infection nor stimulation with VLPs induced significant changes in the expression of these markers. The exception was *MSR1*, which was significantly downregulated in SARS-CoV-2 infected MDMs at 12 and 36 hpi (Fig 3A). These findings suggest that SARS-CoV-2 infection favors a pro-inflammatory profile in MDMs, consistent with the observed upregulation of associated co-stimulatory molecules (Fig 3A). Indeed, SARS-CoV-2 infection induced a significant increase in the expression of TLRs involved in viral PAMP recognition, including *TLR2* and *TLR8*, with a peak at 12 hpi (Fig 3B). In contrast, *TLR3* and *TLR7* mRNA expressions were downregulated from early time points post-infection (Fig 3B), suggesting that viral infection could suppress endosomal viral RNA-sensing pathways.

Both SARS-CoV-2 infection and stimulation with VLPs promoted mRNA expression of cytoplasmic viral RNA sensors, including *DDX58/RIG-I*, *IFIH1/MDA5*, and members of DExD/H-box helicases family (*DHX58/LGP2*, *DDX60*, *DDX60L*), as well as *IFIT1–3,* and double-stranded RNA-activated protein kinase R (*EIF2AK2/PKR)* (Fig 3B). Moreover, MDMs stimulated with VLPs, and to a greater extent those infected with SARS-CoV-2, exhibited mRNA expression of NF-κB complex components, including *NFKB1*, *RELA*, *NFKB2*, *RELB*, *IKBIA/IKBA*, and *IKBIZ/IKB*Z from early time points post-infection (Fig 3C). Concomitantly, high expression of canonical NF-κB target genes was also observed, including inflammatory cytokines (*TNF*, *IL1B*, *CXCL8/IL8*, *IL12B*, *IL23A*), growth factors (*CSF1/M-CSF*, *CSF2/GM-CSF*), chemokines (*CXCL1*–*3*, *CXCL5*, *CCL3*, *CCL4*), and cyclooxygenase-2 (*PTGS2/COX2*; Fig 3C). These results confirm that recognition of SARS-CoV-2 structural proteins, such as Spike and Envelope, is sufficient to trigger NF-κB activation and downstream inflammatory gene expression in human MDMs. However, this response was markedly stronger following infection with SARS-CoV-2 instead of VLPs, suggesting that sensing of the viral genome and/or replication intermediates is critical for amplifying the inflammatory response.

### Robust activation of IL-6 signaling pathway in macrophages requires infectious SARS-CoV-2, thereby recapitulating the monocyte transcriptional signature observed in COVID-19 patients

Like the findings observed in monocytes from COVID-19 patients (Fig 2A), SARS-CoV-2 infection of MDMs induced a highly significant upregulation of IL-6 and key components of its signaling pathway, including *IL6R*, *JAK1*, *JAK2*, *STAT3*, and *SOCS3*, with expression peaks at 4 and 12 hpi (Fig 3D). However, this induction was not observed in VLPs-stimulated MDMs (Fig 3D). These results were consistent with transcriptional priming of canonical IL-6 signaling target genes, including *JAK3*, *MYC*, *BCL2L1*, *MCL1*, *MMP2*, Vascular endothelial growth factor A (*VEGFA)*, and *IL10* (Fig 3D). The transcriptional induction of these genes in SARS-CoV-2-infected MDMs suggests the activation of the IL-6 signaling pathway, closely matching the profile observed in monocytes from COVID-19 patients (Fig 2A and 2B). By contrast, VLP treatment of MDMs increased IL-6 mRNA expression but was insufficient to promote robust and sustained transcriptional activation of the downstream signaling pathway (Fig 3D). These observations suggest that high-level IL-6 production and full pathway activation in MDMs depend on recognition of viral particles and/or viral replication intermediates.

### SARS-CoV-2 infection is associated with suppression of Vitamin D Receptor signaling in human macrophages

To determine whether *in vitro* SARS-CoV-2 infection modulates the expression of components of the VD/VDR pathway in MDMs, we analyzed the transcriptional landscape of both SARS-CoV-2-infected and VLP-stimulated MDMs. In contrast to the findings observed in monocytes from COVID-19 patients (Fig 2C), SARS-CoV-2-infected MDMs exhibited downregulation of *VDR* and *CYP2R1*, accompanied by a modest induction of *RXRA* at 4 hpi (Fig 3E), suggesting early repression of VDR-dependent signaling in virus-infected MDMs. Moreover, SARS-CoV-2-infected MDMs showed reduced expression of canonical VDR target genes, including *CAMP/LL37* and *IRF5*, at 12 and/or 36 hpi (Fig 3E), indicating suppression of VDR signaling during infection. Furthermore, infected MDMs upregulated the expression of *CYP27B1*, *CD14*, and *CD274/PD-L1* (Fig 3E). Notably, these alterations in VDR expression and downstream signaling were not observed in VLP-stimulated MDMs, except for *CYP27B1* and *CD274/PD-L1* (Fig 3E), suggesting that suppression of the VDR pathway could depend on viral replication and/or expression of non-structural proteins during active infection.

### Functional validation of VD-mediated modulation of SARS-CoV-2-induced inflammatory responses in human monocytes and macrophages

To validate the functional role of vitamin D in SARS-CoV-2 infection, we performed a functional assay in human mononuclear phagocytes derived from a cell line. To determine which of the most widely used human promonocytic cell lines in research, U937 and THP-1 [49,60,61], might represent a better model for studying the IL-6-dependent cytokine storm induced by SARS-CoV-2 infection, we reanalyzed the bulk RNA-seq dataset GSE308204 (GEO) [49]. This dataset was generated from U937 and THP-1 cells differentiated into macrophages with 10 ng/mL PMA for 48 hours. Initially, we validated the immunophenotype of both macrophage models, observing that both cell populations express classical myeloid/monocytic markers such as *CD68*, *CD14*, *ITGAM/CD11b*, and *CCR1*. However, the transcriptional expression of these genes was significantly higher in U937 cells compared to THP-1 cells (S1A). On the other hand, THP-1 cells expressed significantly higher levels of *S100A8* than U937 cells but did not express *FCGR3A/CD16A* (S1A). Further, we observed that U937 cells, unlike THP-1 cells, showed high and significant expression of classical macrophage markers, including phagocytosis receptors such as *CD163*, *MSR1*, *MARCO*, and *FCGR2A/CD32A*, as well as costimulatory molecules like *CD86* (S1B). This suggests that U937 cells exhibit an immunophenotype more closely resembling primary macrophages compared to THP-1 cells. Notably, both cell lines lacked *HLA-DR* expression (S1B), indicating a functional deficiency in antigen-presenting capacity for both cell lines.

We next assessed the ability of both cell lines to detect SARS-CoV-2 PAMPs. U937 cells expressed significantly higher levels of pattern recognition receptors involved in sensing viral infection, including *TLR4*, *DDX58/RIG-I*, *IFIH1/MDA5*, and *EIF2AK2/PKR* (S1C), compared to THP-1 cells. However, both cell lines expressed similar levels of *TLR2* mRNA and showed very low or no expression of TLRs involved in sensing viral genome, including *TLR3*, *TLR7*, and *TLR8* (S1C). These findings suggest that both cell lines are deficient in key innate mechanisms to sensing RNA virus infection. Finally, we validated the ability of both cell lines to respond to vitamin D. Both cell lines expressed all components involved in VD3 biosynthesis and signaling through the VDR (S1D). However, U937 cells expressed significantly higher levels of *VDR* and *CYP2R1*, whereas THP-1 cells exhibited significantly higher levels of *RXRA* (S1D). Both cell lines expressed VDR target genes, including *LYZ* and *IRF5* (S1D).

Taken together, our transcriptomic characterization of macrophages derived from U937, or THP-1 cells, shows that both models possess all the critical components required to study macrophage inflammatory responses to SARS-CoV-2 infection. However, given their closer macrophage-like phenotype, higher expression of PRRs involved in detecting viral infection, and greater expression of VDR components and the VD3 biosynthesis machinery, we conclude that U937 cells represent a better experimental model for the study of macrophage inflammatory responses to SARS-CoV-2 infection and the influence of vitamin D in controlling pathological inflammatory responses, as compared to THP-1 cells.

Next, we validated the efficiency of the PMA-based differentiation method for generating macrophages from U937 cells. In this case, U937 cells were treated with or without 100 nM PMA for 3 days at 37°C in a 5% CO_2_ atmosphere to obtain U937-derived macrophages and U937-derived monocytes, respectively. The cell morphotypes and immunophenotype of both models were then confirmed by microscopy and flow cytometry, respectively. As previously described [61], we confirmed that PMA treatment of U937 cells leads to increased cell adhesion and morphological changes, characterized by an increase in cytoplasmic projections (S1E). Furthermore, we confirmed that PMA treatment resulted in increased surface expression of CD14 and CD11b on U937-derived macrophages compared to U937-derived monocytes (S1F). To further confirm the ability of U937 cells to respond to vitamin D, U937 cells were differentiated into macrophages in the presence of 100 nM PMA and 10 nM Calcitriol for 3 days. Under these conditions, we observed that these macrophages expressed higher levels of CD11b, a well-known VDR target gene, compared to cells differentiated with PMA alone (S1F). This finding confirms the ability of U937 cells to respond efficiently to vitamin D treatment.

**Fig 4:**
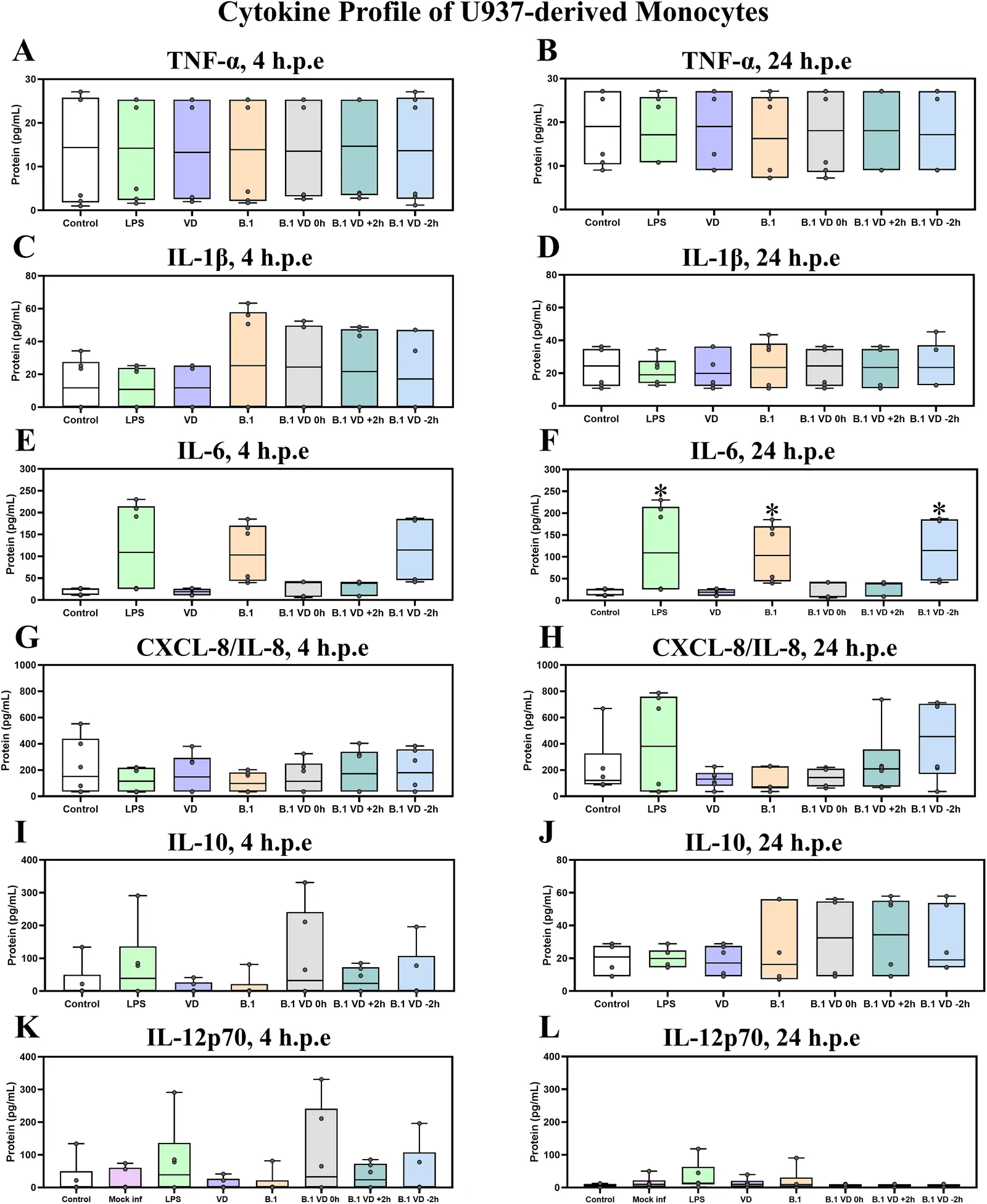
Vitamin D modulates interleukin-6 production in human monocytes challenged with SARS-CoV-2. Human U937-derived monocytes were exposed or not to UV‑light‑inactivated SARS‑CoV‑2 particles (lineage B.1, MOI 0.5) and stimulated or not with 10 nM calcitriol (VD) at three time points: simultaneously with viral challenge (0 h), 2 hours before (−2 h), or 2 hours after (+2 h) viral challenge. Cells treated with 100 ng/mL LPS served as positive control for immune activation. Culture supernatants were collected at 4 and 24 h.p.s, and concentrations of TNF‑α, IL‑1β, IL‑6, CXCL‑8/IL‑8, IL‑10, and IL‑12p70 were measured using a human inflammatory cytokine cytometric bead array (CBA) kit. Cytokine concentrations are shown for: TNF‑α at 4 h.p.s (A) and 24 h.p.s (B); IL‑1β at 4 h.p.s (C) and 24 h.p.s (D); IL‑6 at 4 h.p.s (E) and 24 h.p.s (F); CXCL‑8/IL‑8 at 4 h.p.s (G) and 24 h.p.s (H); IL‑10 at 4 h.p.s (I) and 24 h.p.s (J); and IL‑12p70 at 4 h.p.s (K) and 24 h.p.s (L). Data represent 2‑3 technical replicates with two independent repetitions per condition, presented as the median with interquartile range. Normality was assessed with the Shapiro–Wilk test, and group comparisons were performed using the Kruskal–Wallis test with Dunn’s post‑hoc test (uncorrected). Significant differences (p < 0.05) between unstimulated control cells and each experimental condition are indicated by an asterisk (*).

Following the functional validation of our experimental models, U937-derived monocytes and macrophages were stimulated with UV-inactivated SARS-CoV-2 from a Colombian clinical isolate, lineage B.1 (B.1), and treated with calcitriol (10 nM; VDR agonist) either 2 h pre-stimulation (B.1 VD -2h), simultaneously (B.1 VD 0h), or 2 h post-stimulation (B.1 VD + 2h). Cells were incubated for 4 or 24 h (S1G). In U937-derived monocytes, stimulation with LPS (positive control) or only stimulated with UV-inactivated SARS-CoV-2 particles (B.1) did not induce significant changes in the production of NF-κB-dependent inflammatory mediators, including TNF-α (Fig 4A, 4B), IL-1β (Fig 4C, 4D), CXCL-8/IL-8 (Fig 4G, 4H), IL-10 (Fig 4I, 4J), and IL-12p70 (Fig 4K, 4L) at either 4 or 24 hours post-exposure. Importantly, IL-6 production was significantly reduced in the conditions B.1 VD 0h or B.1 VD + 2h, but not in B.1 VD -2h, and this effect was observed exclusively at 24 hps (Fig 4F). These findings demonstrate that VD exerts a potent immunomodulatory effect by suppressing IL-6 production in monocyte-like cells stimulated with UV-inactivated SARS-CoV-2.

**Fig 5:**
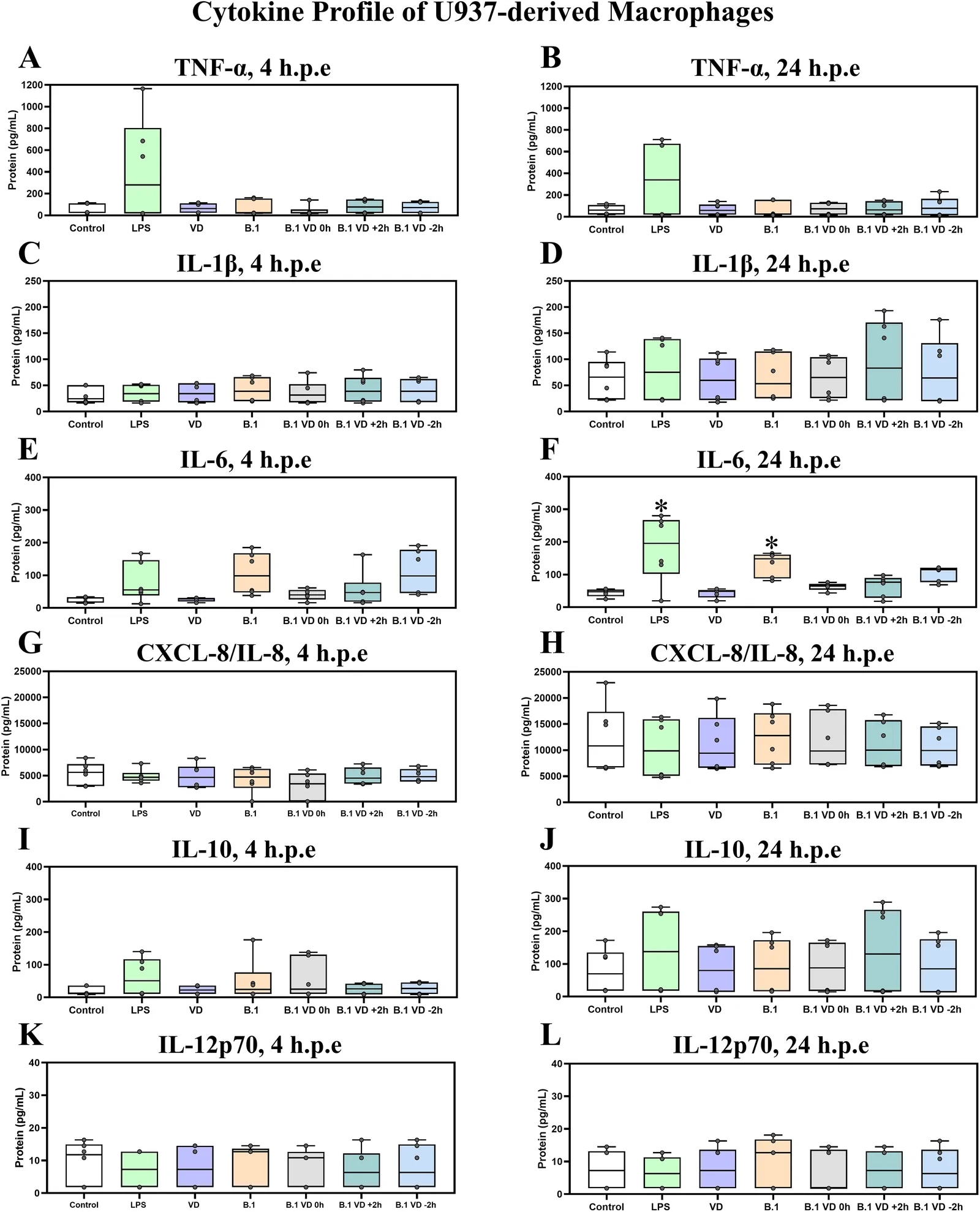
Vitamin D modulated interleukin-6 production in human macrophages challenged with SARS-CoV-2. Human U937-derived macrophages (stimulated with 100 ng/mL PMA for 72h) were exposed or not to UV‑light‑inactivated SARS‑CoV‑2 particles (lineage B.1, MOI 0.5) and stimulated or not with 10 nM calcitriol (VD) at three time points: simultaneously with viral challenge (0 h), 2 hours before (−2 h), or 2 hours after (+2 h) viral challenge. Cells treated with 100 ng/mL LPS served as positive control for immune activation. Culture supernatants were collected at 4 and 24 h.p.s, and concentrations of TNF‑α, IL‑1β, IL‑6, CXCL‑8/IL‑8, IL‑10, and IL‑12p70 were measured using a human inflammatory cytokine cytometric bead array (CBA) kit. Cytokine concentrations are shown for: TNF‑α at 4 h.p.s (A) and 24 h.p.s (B); IL‑1β at 4 h.p.s (C) and 24 h.p.s (D); IL‑6 at 4 h.p.s (E) and 24 h.p.s (F); CXCL‑8/IL‑8 at 4 h.p.s (G) and 24 h.p.s (H); IL‑10 at 4 h.p.s (I) and 24 h.p.s (J); and IL‑12p70 at 4 h.p.s (K) and 24 h.p.s (L). Data represent 2‑3 technical replicates with two independent repetitions per condition, presented as the median with interquartile range. Normality was assessed with the Shapiro–Wilk test, and group comparisons were performed using the Kruskal–Wallis test with Dunn’s post‑hoc test (uncorrected). Significant differences (p < 0.05) between unstimulated control cells and each experimental condition are indicated by an asterisk (*).

Similarly to what was observed in U937-derived monocytes, U937-derived macrophages stimulated with UV-inactivated SARS-CoV-2 (B.1) did not exhibit significant changes in the production of NF-κB-dependent inflammatory mediators, including TNF-α (Fig 5A, 5B), IL-1β (Fig 5C, 5D), CXCL-8/IL-8 (Fig 5G, 5H), IL-10 (Fig 5I, 5J), and IL-12p70 (Fig 5K, 5L), at either 4 or 24 hps. Notably, the IL-6 production was remarkably reduced upon calcitriol treatment administered simultaneously with UV-inactivated SARS-CoV-2 stimulation (B.1 VD 0h) or 2 hours after (B.1 VD + 2h) (Fig 5F). These results confirm that VD exerts a potent immunomodulatory effect by efficiently suppressing SARS-CoV-2-induced IL-6 production in macrophage-like cells. Our functional assay demonstrates that VD exhibits significant immunomodulatory properties, suppressing SARS-CoV-2-induced IL-6 production in both human monocyte- and macrophage-like cells.

## Discussion

This study provides a comprehensive analysis of the innate immune response of primary human mononuclear phagocytes during SARS-CoV-2 infection by integrating *ex vivo* transcriptomic data from COVID-19 patients with *in vitro* functional models. Our findings underscore the central and dual roles of monocytes, macrophages, and mDC in COVID-19, as these cells are pivotal for initiating antiviral defenses while also contributing to the dysregulated inflammation that characterizes severe disease [8,14–16]. Importantly, we reveal dysregulation of the Vitamin D-VDR signaling pathway in these cells upon SARS-CoV-2 infection and demonstrate that treatment with calcitriol effectively suppresses a key pathological driver of severe COVID-19, namely SARS-CoV-2-induced IL-6 hyperproduction [5,10,11].

Our initial transcriptional profiling confirmed that SARS-CoV-2 infection significantly modulates the immunophenotype of circulating monocytes and mDCs from COVID-19 patients. The consistent downregulation of *HLA-DRA* on both cell types, regardless of viremia status, could be related to reports describing impaired antigen presentation in severe COVID-19 [62,63]. This finding was recapitulated in the *in vitro* macrophage infection model, in which infectious SARS-CoV-2, but not VLPs, suppressed *HLA-DRA* expression. These results suggest a viral strategy to evade adaptive immunity by dampening MHC class II-mediated antigen presentation, potentially through viral proteins that interfere with IFN signaling and/or CIITA transcription [8,22,64–66].

**Fig 6:**
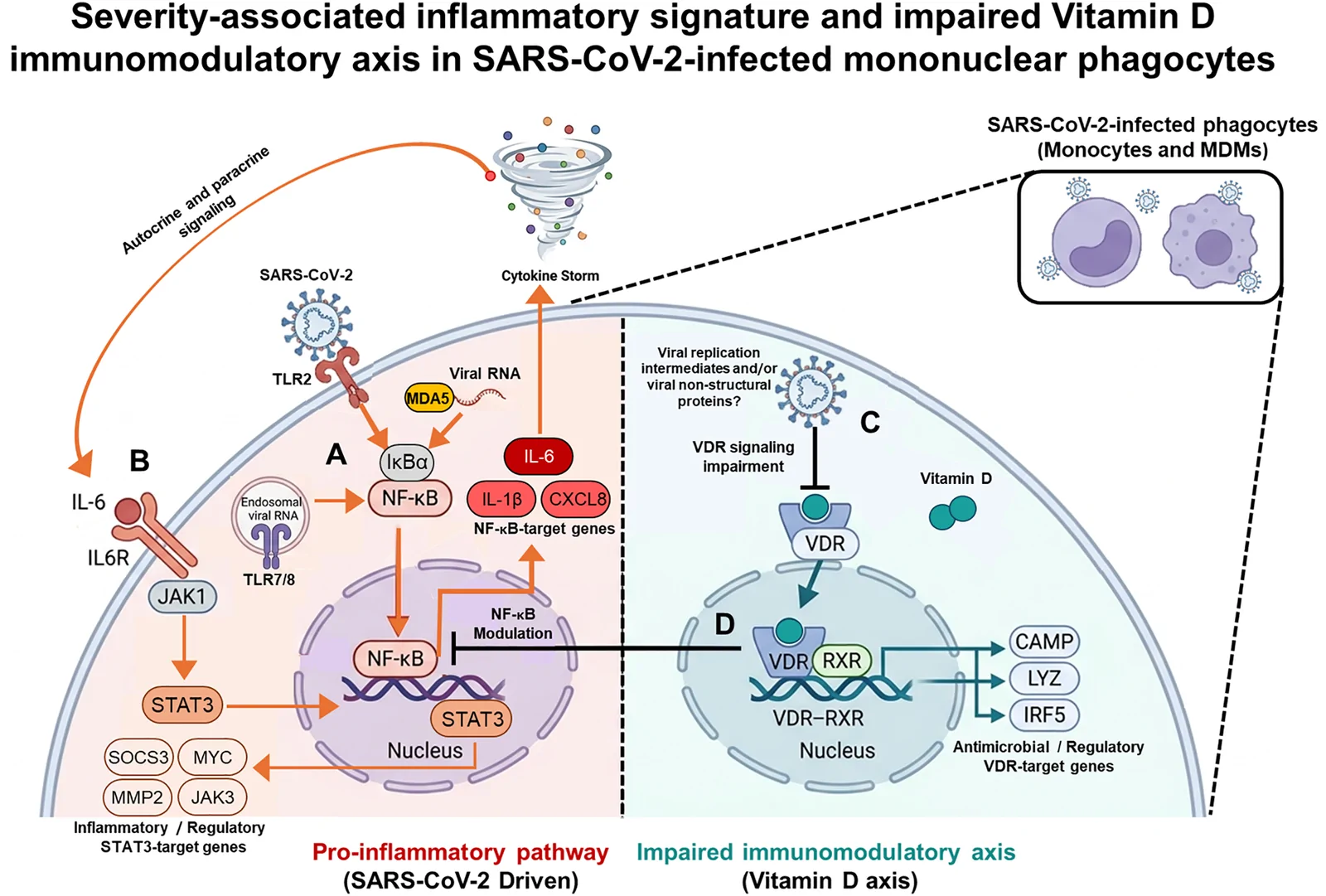
Severity-associated inflammatory signature and impaired Vitamin D immunomodulatory axis in SARS-CoV-2-infected mononuclear phagocytes. (A) SARS-CoV-2 infection induces hyperactivation of the PRR/NF-κB/IL-6 inflammatory axis. (B) Amplification of inflammation through autocrine and paracrine IL-6 signaling and induction of pro-inflammatory and regulatory STAT3-target genes. (C) Viral-mediated impairment of the immunomodulatory Vitamin D/VDR signaling pathway. (D) Pharmacological activation of VDR by calcitriol (active Vitamin D) attenuates virus-induced IL-6 production through modulation of NF-κB activation.

A core finding of our work is the cell population and disease-severity-specific activation of PRRs and the NF-κB pathway (Fig 6A). We observed that monocytes from COVID-19 patients exhibited significant upregulation of *TLR2* and *TLR8*, whereas macrophages *in vitro* infected showed an early induction of these receptors, accompanied by a robust downregulation of *TLR3* and *TLR7*. This pattern suggests a strategic viral modulation of innate sensing. The upregulation of *TLR2* and *TLR8*, sensors for viral structural glycoproteins and ssRNA, respectively, is consistent with their established role in driving NF-κB-dependent pro-inflammatory cytokine production in response to SARS-CoV-2 [6,18,67,68]. On the other hand, downregulation of endosomal TLRs (*TLR3* and *TLR7*) could represent a viral immune evasion mechanism aimed at blunting type I interferon response, a characteristic of severe COVID-19 [65–67]. Importantly, monocytes from patients with moderate COVID-19 exhibited significantly higher levels of *IFIH1/MDA5* than those from healthy donors or patients with severe disease, suggesting that MDA5 activation in moderate cases could confer protection against progression to severe COVID-19. Also, the stronger inflammatory response of MDMs to SARS-CoV-2 *in vitro* infection compared to VLPs underscores the requirement for viral replication and/or non-structural proteins in amplifying inflammation.

Hyperactivation of the NF-κB pathway drives excessive IL-6 production, a key mediator of COVID-19 severity [5,10,11]. Elevated IL-6 levels correlate with higher Brixia scores on chest X-rays, reflecting greater lung injury [69]. This reinforces IL-6 as a critical biomarker and therapeutic target in severe COVID-19. Our data delineates a coherent pathogenic cascade initiated by SARS-CoV-2 infection of monocytes and macrophages, which promotes PRRs engagement (TLR2, TLR8, RIG-I, MDA-5) and NF-κB activation (Fig 6A). This, in turn, drives transcriptional upregulation of *IL6* and key components of its signaling axis (*IL6R*, *JAK1/2*, *STAT3*, and *SOCS3*), thereby promoting autocrine and paracrine signaling loops that further amplify the expression of inflammatory target genes (*JAK3*, *MYC*, *BCL2L1*, *MMP2*) (Fig 6B). This inflammatory circuit was consistently evidenced in monocytes derived from COVID-19 patients, and in SARS-CoV-2-infected MDMs. Notably, the magnitude of this response was markedly stronger in MDMs during active SARS-CoV-2 infection, suggesting that direct recognition of the viral genome and/or replication-associated intermediates is critical for amplifying inflammatory signaling. Importantly, we found that mDCs displayed a more restricted inflammatory profile, characterized by the induction of selected NF-κB target genes, including *IL1B* and *CXCL8/IL8*, but lacking activation of the full IL-6 signaling axis. Collectively, these findings emphasize that monocytes and macrophages, rather than mDCs, are the primary cellular drivers of the IL-6-dependent cytokine storm in COVID-19, a distinction with important implications for the development of targeted immunomodulatory therapies.

The most novel and translational aspect of our research lies in investigating the vitamin D-VDR pathway. Contrary to the simplistic hypothesis that vitamin D deficiency alone worsens COVID-19 outcomes [39,55], our data point out that SARS-CoV-2 infection itself actively dysregulates this immunomodulatory system (Fig 6C). In monocytes derived from COVID-19 patients, we observed an unexpected increase in *VDR* and *RXRA* expression, accompanied by a striking suppression of canonical VDR target genes (*LYZ, IRF5*, *CAMP/LL37*, and *CD14*). This transcriptional pattern indicates a dysregulation of VDR signaling in which the receptor is present, but its downstream transcriptional program is functionally silenced [70,71]. This phenomenon was even more pronounced *in vitro*, where SARS-CoV-2 infection of MDMs directly downregulated *CYP2R1* and *VDR*, as well as their target genes. Notably, this repression was infection-dependent but independent of NF-κB and SOCS3 activation, as it was not observed following stimulation with VLPs, which also induce both NF-κB and *SOCS3*. This suggests that replication-associated intermediates and/or viral non-structural proteins are involved in suppressing this critical anti-inflammatory pathway. By dismantling the VDR-mediated brake on inflammation, SARS-CoV-2 may thereby facilitate the unchecked NF-κB activation and IL-6 responses observed in our study (Fig 6C) [23,26,72]. Importantly, monocytes from patients with moderate COVID-19 exhibited significantly higher *CYP27B1* expression, a key enzyme in the VD biosynthesis pathway [27], suggesting that increased CYP27B1 levels in monocytes may enhance VDR signaling capacity and act as a protective factor against progression to severe disease.

To functionally validate this immunomodulatory mechanism, it was essential to establish an in vitro model that faithfully recapitulates the key features of primary human mononuclear phagocytes. Our comparative transcriptomic analysis of U937- and THP-1-derived macrophages revealed that U937 cells closely approximate the primary macrophage immunophenotype. Specifically, U937 cells exhibited significantly higher expression of classical myeloid markers, phagocytic receptors, and costimulatory molecules. Furthermore, U937 cells expressed higher levels of pattern recognition receptors critical for sensing viral infections, positioning them as a more suitable model for studying SARS-CoV-2 sensing despite both lines showing low expression of endosomal *TLR3/7/8*. Crucially, regarding vitamin D biology, while both cell lines expressed the complete VD3 biosynthesis and VDR signaling machinery, U937 cells demonstrated significantly higher expression of *VDR* and *CYP2R1*, key components for vitamin D responsiveness. Together, these results establish U937-derived macrophages as a suitable experimental model to investigate the interplay between SARS-CoV-2 and vitamin D-mediated immunomodulation.

Our simplified functional monocyte/macrophage assay, which directly assessed the therapeutic implications of this finding, responded to viral structural proteins such as S and E present in the envelope of inactivated viral particles. However, it could not evaluate the mechanism by which SARS-CoV-2 replication-associated intermediates disrupt VDR signaling. Nevertheless, we observed that both pre- and post-treatment with calcitriol potently inhibited SARS-CoV-2-induced IL-6 production in U937-derived monocytes and macrophages (Fig 6D). This effect was specific, as the production of other cytokines such as TNF-α or IL-1β was largely unaffected in this model. This result matches with known mechanisms of vitamin D action, which include direct interference with NF-κB binding to the IL-6 promoter [32,72], induction of the negative regulator SOCS3 [73], and promotion of a more tolerogenic cell phenotype [74]. Our data suggest that vitamin D does not broadly suppress all immunity but rather selectively tempers a key pathological axis (Fig 6D). This is consistent with meta-analyses reporting that vitamin D supplementation is associated with reduced severity and mortality in COVID-19, particularly among individuals with vitamin D deficiency [39]. Further, in individuals with abnormal glucose homeostasis, vitamin D supplementation significantly reduces circulating levels of CRP, IL-6, and TNF-α [75]. Additionally, vitamin D supplementation significantly promotes wound healing in diabetic foot ulcer patients, reducing wound area and depth [76]. This anti-inflammatory and tissue repair effects support vitamin D’s potential therapeutic role in COVID-19, where cytokine storm and hyperinflammation are key drivers of tissue damage and disease severity.

Of note, pre-treatment with calcitriol (2h before stimulation) did not yield the same effect, suggesting that the timing of vitamin D administration relative to viral encounter is critical for its anti-inflammatory action. These findings directly support our *in vivo* observations: by counteracting the VDR pathway dysregulation induced by SARS-CoV-2, exogenous vitamin D (calcitriol) can restore the VDR-mediated brake on inflammation, specifically limiting the pathological IL-6 response without broadly suppressing other NF-κB-dependent mediators. Thus, our results reconcile the protective role of adequate vitamin D status with the virus-driven disruption of VDR signaling, highlighting a narrow therapeutic window wherein timely calcitriol supplementation may prevent progression to severe disease by targeting IL-6-driven cytokine storm.

### Perspectives

While our in vitro findings with calcitriol treatment are encouraging, further studies are necessary to better understand the role of PRR/NF-kB/IL-6 inflammatory axis and the active suppression of Vitamin D/VDR signaling pathway in SARS-CoV-2 infection. Additionally, given the intrinsic limitations of the U937 model regarding TNF‑α and IL‑1β production, future work should assess the inflammatory potential of SARS‑CoV‑2‑conditioned media from primary human macrophages or monocytes on endothelial cells to fully elucidate the NF-kB‑driven vascular pathology in COVID‑19.

Moreover, underscore the need for well-designed clinical trials to define optimal dosing and timing of vitamin D or its analogs as adjunctive immunomodulators in COVID-19. Future studies should identify the viral determinants driving VDR suppression in monocytes and macrophages and assess whether restoring VDR function enhances antigen presentation and mitigates broader immune dysregulation in severe disease. Such strategies could offer a multifaceted therapeutic approach.

### Limitations

Several limitations should be acknowledged. First, it was not determined whether MDMs were productively infected by SARS-CoV-2 and produced infectious progeny. Thus, while transcriptional responses indicate viral sensing, we cannot distinguish those driven by active replication from those driven by abortive infection or by recognition of viral genome/viral proteins.

Second, the analysis was based on a single ancestral SARS-CoV-2 strain. Findings may not extend to emerging variants with altered tropism, replication capacity, or immunomodulatory profiles, which could differentially affect the vitamin D/VDR axis.

Third, while the U937 cell line enabled mechanistic interrogation of VD effects, it exhibits functional TLR deficits and impaired TNFα/IL-1β production. Moreover, VDR signaling was not explored in this study. Validation in primary monocytes and macrophages from multiple donors is needed to address inter-individual variability and physiological relevance.

Fourth, our *ex vivo* analyses were cross-sectional, precluding causal inference and temporal resolution of VDR–inflammation dynamics during infection.

Finally, we focused on transcriptional and cytokine readouts without direct functional assays (e.g., phagocytosis, antigen presentation). Future studies integrating functional endpoints, longitudinal sampling, and variant-specific models are required.

## Conclusion

This study establishes that SARS-CoV-2 infection induces profound dysregulation in primary human mononuclear phagocytes, driving immunopathology through two interconnected mechanisms: hyperactivation of the PRR/NF-κB/IL-6 inflammatory axis, and active suppression of the anti-inflammatory Vitamin D/VDR signaling pathway (Fig 6). Importantly, we demonstrate that pharmacological activation of VDR by calcitriol potently suppresses virus-induced IL-6 production in both human monocyte and macrophage-like cells. Collectively, these findings identify VDR signaling dysregulation as a key contributor to cytokine storm characteristic of severe COVID-19, and support vitamin D-mediated VDR activation as a rational immunomodulatory strategy to attenuate pathological inflammation during SARS-CoV-2 infection.

### Use of Artificial Intelligence Tools statement

Artificial intelligence (AI)-assisted tools were used in a limited capacity during the preparation of this manuscript. Specifically, ChatGPT and DeepSeek were used to assist in improving the clarity, grammar, and overall readability of the text, while Grammarly was used for language editing. These tools were employed solely to refine the expression of the authors’ original writing and did not contribute to the generation or interpretation of scientific content. In addition, ChatGPT was used to assist in generating an initial prompt for the conceptual design of the graphical model presented in Fig 6 and S1G. This prompt was subsequently used in the generative AI system Gemini to produce a preliminary visual backbone of the graphical summary. The resulting output served only as an initial conceptual template and was extensively modified, curated, and finalized manually by the authors using PowerPoint to ensure scientific accuracy, appropriate representation of the underlying biological concepts, and consistency with the data presented in the manuscript.

All scientific ideas, experimental design, data analysis, interpretations, and conclusions are the sole responsibility of the authors.

## Supporting information

S1 Fig Characterization of U937-derived monocytes and macrophages models. We reanalyzed the publicly available RNA-Seq dataset GSE308204 (GEO; see Methods and Table 1), with gene expression quantified as Transcripts Per Million (TPM). Panels A–D show the mRNA abundance of (A) monocyte and dendritic cell markers, (B) macrophage markers, (C) pattern recognition receptors (PRRs) involved in SARS-CoV-2 sensing, and (D) components of the VDR signaling pathway. Data are presented as mean ± SEM. Normality was assessed using the Shapiro–Wilk test, and group comparisons were performed using Student’s t-test. Significant differences (p < 0.05) between U937- and THP-1-derived macrophages are indicated by asterisks (*). U937-derived monocytes and macrophages (stimulated with 100 ng/mL PMA for 72 h) were visualized by phase-contrast microscopy. (E) Representative images illustrating the morphological features of U937 cells are shown. (F) Surface expression of CD14 and CD11b on U937-derived monocytes, and macrophages differentiated in the presence or absence of 10 nM calcitriol, were assessed by flow cytometry. Gray histograms represent the corresponding isotype-control staining. Data is representative of at least two independent experiments. (G) Schematic representation of the experimental strategy used to evaluate the immunomodulatory effect of calcitriol (VD) on the SARS-CoV-2-induced cytokine storm.
(TIF)

S1 File PLOS human participants research checklist. (PDF)
