# Supplementary material for: SARS-CoV-2 infection induces functional impairment of vitamin D receptor signaling to drive interleukin 6-dependent hyperinflammation in mononuclear phagocytes
Source: PLoS One. 2026 Sep 11;21(9):e0357030. doi: 10.1371/journal.pone.0357030 (PMC13567757; doi:10.1371/journal.pone.0357030)
Supplement: Peer review history

# Peer review: Decision Letter 0

**DOI:** 10.1371/journal.pone.0357030.r001

**Authors:** Muhammad Iqhrammullah

24 May 2026

Dear Dr. Navas,

Thank you for submitting your manuscript to PLOS ONE. After careful consideration, we feel that it has merit but does not fully meet PLOS ONE’s publication criteria as it currently stands. Therefore, we invite you to submit a revised version of the manuscript that addresses the points raised during the review process.

Please submit your revised manuscript by Jul 08 2026 11:59PM. If you will need more time than this to complete your revisions, please reply to this message or contact the journal office at plosone@plos.org. When you're ready to submit your revision, log on to https://www.editorialmanager.com/pone/ and select the 'Submissions Needing Revision' folder to locate your manuscript file.

- A letter that responds to each point raised by the academic editor and reviewer(s). You should upload this letter as a separate file labeled 'Response to Reviewers'.
- A marked-up copy of your manuscript that highlights changes made to the original version. You should upload this as a separate file labeled 'Revised Manuscript with Track Changes'.
- An unmarked version of your revised paper without tracked changes. You should upload this as a separate file labeled 'Manuscript'.

If you would like to make changes to your financial disclosure, please include your updated statement in your cover letter. Guidelines for resubmitting your figure files are available below the reviewer comments at the end of this letter.

If applicable, we recommend that you deposit your laboratory protocols in protocols.io to enhance the reproducibility of your results. Protocols.io assigns your protocol its own identifier (DOI) so that it can be cited independently in the future. For instructions see: https://journals.plos.org/plosone/s/submission-guidelines#loc-laboratory-protocols. Additionally, PLOS ONE offers an option for publishing peer-reviewed Lab Protocol articles, which describe protocols hosted on protocols.io. Read more information on sharing protocols at https://plos.org/protocols?utm_medium=editorial-email&utm_source=authorletters&utm_campaign=protocols.

As the corresponding author, your ORCID iD is verified in the submission system and will appear in the published article. PLOS supports the use of ORCID, and we encourage all coauthors to register for an ORCID iD and use it as well. Please encourage your coauthors to verify their ORCID iD within the submission system before final acceptance, as unverified ORCID iDs will not appear in the published article. *Only* the individual author can complete the verification step; PLOS staff *cannot* verify ORCID iDs on behalf of authors.

We look forward to receiving your revised manuscript.

Kind regards,

Muhammad Iqhrammullah, Ph.D

Academic Editor

PLOS One

Journal Requirements:

When submitting your revision, we need you to address these additional requirements.

1. Please ensure that your manuscript meets PLOS ONE's style requirements, including those for file naming. The PLOS ONE style templates can be found at

https://journals.plos.org/plosone/s/file?id=wjVg/PLOSOne_formatting_sample_main_body.pdf and

https://journals.plos.org/plosone/s/file?id=ba62/PLOSOne_formatting_sample_title_authors_affiliations.pdf

2. Please include captions for your Supporting Information files at the end of your manuscript, and update any in-text citations to match accordingly. Please see our Supporting Information guidelines for more information: http://journals.plos.org/plosone/s/supporting-information.

3. If the reviewer comments include a recommendation to cite specific previously published works, please review and evaluate these publications to determine whether they are relevant and should be cited. There is no requirement to cite these works unless the editor has indicated otherwise.

[Note: HTML markup is below. Please do not edit.]

Reviewers' comments:

Reviewer's Responses to Questions

**Comments to the Author**

1. Is the manuscript technically sound, and do the data support the conclusions?

Reviewer #1: Partly

Reviewer #2: Partly

**********

2. Has the statistical analysis been performed appropriately and rigorously?

Reviewer #1: Yes

Reviewer #2: Yes

**********

3. Have the authors made all data underlying the findings in their manuscript fully available?

The PLOS Data policy

Reviewer #1: Yes

Reviewer #2: No

**********

4. Is the manuscript presented in an intelligible fashion and written in standard English?

Reviewer #1: Yes

Reviewer #2: Yes

**********

Reviewer #1: 1.(Abstract): The abstract mentions JAK1 as a component of IL-6 signaling, but in Results section 3.6 and Figure 3D, it is stated that both JAK1 and JAK2 are induced in SARS-CoV-2-infected MDMs. Please re-check and standardize.

2.(Introduction, line 130): The term "VLPs" appears for the first time without a definition. Please define it as "virus-like particles (VLPs)" at its first appearance in the Introduction, then use the abbreviation VLPs in subsequent sections.

3.(Methods): Revision is required for the Ethics statement. There is no need to claim an emergency policy waiver of approval. Simply state that this study is a re-analysis of publicly available data, and that the original studies obtained proper ethical approval. No new human samples were collected by the authors.

4.(Results, section 3.3): The text mentions MMP2 as an IL-6 signaling target gene in monocytes. However, Figure 2B shows MMP9. Please verify and standardize between the text and the figure. If the correct data is MMP9, revise the text to MMP9. If the correct data is MMP2, revise the figure.

5.(Discussion): The discussion states that VDR suppression depends on replication-associated intermediates, but the functional assay used UV-inactivated virus (non-replicating). Please revise the discussion to reflect that the inactivated model cannot test this mechanism.

6.(Discussion): In the Discussion section, where the inhibitory effect of calcitriol on SARS-CoV-2-induced IL-6 production is interpreted as a potential immunomodulatory strategy, the current supporting references are predominantly based on mechanistic in vitro or transcriptomic data. It is suggested to strengthen this interpretation by citing https://pubmed.ncbi.nlm.nih.gov/34126229/, a systematic review and meta-analysis of 38 RCTs demonstrating that vitamin D supplementation significantly reduces IL-6 and CRP levels across diverse clinical conditions, and https://doi.org/10.52225/narrax.v1i3.104, a pooled analysis of RCTs reporting that calcitriol modulates inflammatory markers through VDR–NF-κB pathway inhibition in macrophages. These references would provide important clinical and translational context to support the broader immunomodulatory conclusion proposed in this section.

7.(Discussion): In the Discussion, where the clinical significance of calcitriol-mediated IL-6 suppression is interpreted, it is recommended to cite https://pmc.ncbi.nlm.nih.gov/articles/PMC11125309/, which demonstrated that elevated IL-6 levels significantly predicted severe radiological lung damage in COVID-19 patients. This clinical evidence strengthens the translational relevance of the in vitro IL-6 suppression findings reported in this manuscript.

Reviewer #2: Reviewer Evaluation

Manuscript Number: PONE-D-26-15930

SARS-CoV-2 Infection Induces Functional Impairment of Vitamin D Receptor Signaling to Drive Interleukin 6-Dependent Hyperinflammation in Mononuclear Phagocytes

General Overview

This study investigates a dual mechanism in severe COVID-19, characterized by an IL6-driven cytokine storm and suppression of the vitamin D signaling pathway in mononuclear phagocytes infected with SARS-CoV-2. The authors integrate transcriptomic reanalysis of in vivo and in vitro data with in vitro validation using the U937 cell line to suggest that calcitriol treatment can reduce IL-6 production. While the findings are compelling and provide a rationale for Vitamin D as an immunomodulatory strategy, several technical improvements are required to enhance the manuscript's rigor and clarity.

Major Comments

1. General

The authors rely on the U937 cell line for functional validation (Lines 94–104; 144–147). It is essential to expand the introduction to justify why this specific cell line is an appropriate model for studying the IL-6-mediated cytokine storm in the context of SARS-CoV-2 infection. The authors should introduce evidence regarding U937 gene and protein expression compared with other cells lines and primary human cells. Refer to PMC9677084.

2. Materials and Methods:

A summary table (Lines 159–168) including key features and metadata for the public datasets (GSE216529 and GSE224845) should be included.

In the differential expression analysis (Line 168), please clarify whether the p-value refers to a raw or adjusted p-value. Additionally, specify which enrichment analysis was conducted within the clusterProfiler package (Line 171).

Clarify the timing of the functional assays (Lines 199–200). The current text does not explicitly define the "pre-stimulation," "simultaneous," and "post-stimulation" windows relative to the SARS-CoV-2 challenge. A diagram and clearer notation would help readers.

3. Results

While IL-6 changes are presented, other cytokines did not show significant alterations in the U937 model. The authors should consider including a functional assay to support their conclusion of an IL-6-driven inflammatory response. This reviewer recognizes that conducting additional experiments at this stage may be challenging; however, an experiment assessing the inflammatory potential of conditioned media from U937-infected cells would significantly strengthen the claims made in this manuscript

While noted as a limitation (Line 519), the lack of VDR signaling target gene validation in the U937 model is a significant gap. The manuscript would be greatly strengthened if the authors could provide data for VDR signaling pathway to claim that impairment of this pathway drives the observed inflammatory response.

4. Figures

The legends for Figures 1–3 contain extensive repetition of dataset descriptions, patient group definitions, sample sizes, statistical procedures, and TPM quantification methodology. This information is re-stated in full in each legend despite being described in the Methods section (including the requested summary table). The authors should consolidate this shared information and replace the redundant text in each legend with a brief cross-reference (e.g., "Data from GSE216529; see Methods"). This would substantially improve readability without any loss of information.

Figure 5 is an excellent diagram that effectively summarizes the findings of this manuscript. However, it would benefit from the removal of IL-1β and CXCL8, as these proteins were not found to be modulated in U937 cells.

Minor Comments and Technical Corrections

1. Abstract and Introduction:

The phrasing in lines 38–39 ("both COVID-19 patient monocytes and in vitro infected MDMs an NF-κB-dependent...") is difficult to follow and should be revised for better flow.

Standardize the use of italics for all gene names, including CYP2R1, CYP27A1 (Line 107), CYP27B1 (Line 108), and CAMP (Line 113).

Capitalize and normalize "Zika" (Line 120) and ensure the term "primary human mononuclear phagocytes" is used consistently (Line 124).

2. Results:

Please ensure strict adherence to official gene symbols throughout the section to maintain consistency, including in figures:

Use ITGAM for CD11b and ITGAX for CD11c (Lines 228–229).

Use DDX58 for RIG-I (Line 247).

Use NFKB1 for NF-κB1 (Line 251) and IL1B for IL-1β (Line 255).

Standardize symbols by removing special characters (e.g., use RXRA instead of RXRα and IL6 instead of IL-6 mRNA).

Correct "it" to "its" (Line 356) and replace "Moreover" with "Furthermore" (Line 378) for better transition.

3. Discussion:

Reduce the emphasis on the PRR/NF-κB activation section (Lines 428–447), as this information is heavily detailed in the Results. Instead, expand the section (Lines 465–483) discussing which aspects of the U937 cell line recapitulate primary human monocyte/macrophage responses, and integrate this more thoroughly with the Vitamin D results.

4. Figures

The legends for Figures 4 and 5 the term "kinetics" used in both legends is misleading. The data are presented as discrete box plots at two independent time points (4 and 24 h.p.s.), not as a continuous time-course. The authors should replace "Kinetics are shown for" with a more accurate phrase such as "Cytokine concentrations at 4 and 24 h.p.s. are shown for"

Line 641 contains a typographical error: "collected at 4 and h.p.s" is missing the second time point and should read "collected at 4 and 24 h.p.s."

Specific Questions for the Authors

The MDM data (GSE224845) involves infectious SARS-CoV-2 (Line 165), whereas the U937 validation uses UV-inactivated particles (Line 198). Did the authors observe any differences in the magnitude of VDR suppression when comparing infectious virus to inactivated particles?

Why did the authors use calcitriol instead of calcidiol for their experiments?

What was the rationale for using U937 cells rather than THP-1, which is a well-documented model for studying vitamin D-mediated effects?

**********

what does this mean?). If published, this will include your full peer review and any attached files.

If you choose “no”, your identity will remain anonymous but your review may still be made public.

**Do you want your identity to be public for this peer review?** For information about this choice, including consent withdrawal, please see our Privacy Policy

Reviewer #1: No

Reviewer #2: No

**********

[NOTE: If reviewer comments were submitted as an attachment file, they will be attached to this email and accessible via the submission site. Please log into your account, locate the manuscript record, and check for the action link "View Attachments". If this link does not appear, there are no attachment files.]

To ensure your figures meet our technical requirements, please review our figure guidelines: https://journals.plos.org/plosone/s/figures

You may also use PLOS’s free figure tool, NAAS, to help you prepare publication quality figures: https://journals.plos.org/plosone/s/figures#loc-tools-for-figure-preparation.

NAAS will assess whether your figures meet our technical requirements by comparing each figure against our figure specifications.

# Author comment: Author response to Decision Letter 1

**DOI:** 10.1371/journal.pone.0357030.r002

16 Jul 2026

Doctor

Muhammad Iqhrammullah, Ph.D

Academic Editor PLOS One

Subject: ONE-D-26-15930

Dear Dr. Iqhrammullah,

I am sending the revised version of the manuscript "SARS-CoV-2 Infection Induces Functional Impairment of Vitamin D Receptor Signaling to Drive Interleukin 6-Dependent Hyperinflammation in Mononuclear Phagocytes" and the response to each one of reviewers’ observations.

I would like to thank the reviewers for their thorough reading of the manuscript and their feedback, which enriched the document, improved the quality and strengthened our analyses and results.

Reviewer #1:

1. (Abstract): The abstract mentions JAK1 as a component of IL-6 signaling, but in Results section 3.6 and Figure 3D, it is stated that both JAK1 and JAK2 are induced in SARS-CoV-2-infected MDMs. Please re-check and standardize.

Answer. We thank the reviewer for their insightful observation. We have now included JAK1/2 components in the abstract, on line 40.

2.(Introduction, line 130): The term "VLPs" appears for the first time without a definition. Please define it as "virus-like particles (VLPs)" at its first appearance in the Introduction, then use the abbreviation VLPs in subsequent sections.

Answer. We thank the reviewer for this observation. The definition of virus-like particles (VLPs) has now been included in the manuscript on line 130.

3.(Methods): Revision is required for the Ethics statement. There is no need to claim an emergency policy waiver of approval. Simply state that this study is a re-analysis of publicly available data, and that the original studies obtained proper ethical approval. No new human samples were collected by the authors.

Answer. We thank the reviewer for their valuable suggestion. The corresponding modifications to the bioethical statement have now been included in lines 141-145 of the revised manuscript.

4.(Results, section 3.3): The text mentions MMP2 as an IL-6 signaling target gene in monocytes. However, Figure 2B shows MMP9. Please verify and standardize between the text and the figure. If the correct data is MMP9, revise the text to MMP9. If the correct data is MMP2, revise the figure.

Answer. We thank the reviewer for this observation. MMP2 is referenced in Figure 2B, as well as in the main text.

5.(Discussion): The discussion states that VDR suppression depends on replication-associated intermediates, but the functional assay used UV-inactivated virus (non-replicating). Please revise the discussion to reflect that the inactivated model cannot test this mechanism.

Answer. We greatly appreciate the reviewer's suggestion. Accordingly, we have clarified the limitations of our experimental model by adding the following paragraph to the manuscript (lines 546-549)

6.(Discussion): In the Discussion section, where the inhibitory effect of calcitriol on SARS-CoV-2-induced IL-6 production is interpreted as a potential immunomodulatory strategy, the current supporting references are predominantly based on mechanistic in vitro or transcriptomic data. It is suggested to strengthen this interpretation by citing https://pubmed.ncbi.nlm.nih.gov/34126229/, a systematic review and meta-analysis of 38 RCTs demonstrating that vitamin D supplementation significantly reduces IL-6 and CRP levels across diverse clinical conditions, and https://doi.org/10.52225/narrax.v1i3.104, a pooled analysis of RCTs reporting that calcitriol modulates inflammatory markers through VDR–NF-κB pathway inhibition in macrophages. These references would provide important clinical and translational context to support the broader immunomodulatory conclusion proposed in this section.

Answer. We thank the reviewer very much for this valuable suggestion. We have now incorporated the new references and the following paragraph into the revised manuscript on lines 559-564:

7.(Discussion): In the Discussion, where the clinical significance of calcitriol-mediated IL-6 suppression is interpreted, it is recommended to cite https://pmc.ncbi.nlm.nih.gov/articles/PMC11125309/, which demonstrated that elevated IL-6 levels significantly predicted severe radiological lung damage in COVID-19 patients. This clinical evidence strengthens the translational relevance of the in vitro IL-6 suppression findings reported in this manuscript.

Answer. We thank the reviewer for the suggestion. The reference has now been included, along with the following paragraph, on lines 491-494 of the revised manuscript

Reviewer #2: Reviewer Evaluation

Major Comments

General

1. The authors rely on the U937 cell line for functional validation (Lines 94–104; 144–147). It is essential to expand the introduction to justify why this specific cell line is an appropriate model for studying the IL-6-mediated cytokine storm in the context of SARS-CoV-2 infection. The authors should introduce evidence regarding U937 gene and protein expression compared with other cells lines and primary human cells. Refer to PMC9677084.

Answer. We thank the reviewer for their thoughtful observations. To address this question, we have generated a new supplementary figure (Figure S1), in which we performed a comparative transcriptomic analysis of macrophages derived from U937 and THP-1 cells. In this analysis, we focused on characterizing the immunophenotype, the profile of PRRs involved in SARS-CoV-2 detection, and the expression of components of the VDR pathway. Our transcriptomic characterization of macrophages derived from U937 or THP-1 cells shows that both models possess all the critical components required to study macrophage inflammatory responses to SARS-CoV-2 infection and the influence of vitamin D in controlling pathological inflammatory responses. However, given their closer macrophage-like phenotype, higher expression of PRRs involved in detecting viral infection, and greater expression of VDR components and the VD3 biosynthesis machinery, we conclude that U937 cells represent a better experimental model for our study compared to THP-1 cells. A detailed description of these results can now be found in lines 387-420 of the revised manuscript.

Materials and Methods:

2. A summary table (Lines 159–168) including key features and metadata for the public datasets (GSE216529 and GSE224845) should be included.

Answer. We thank the reviewer for the suggestion. The new Table 1, containing the transcriptome metadata, has now been included in the Materials and Methods section on line 162.

3. In the differential expression analysis (Line 168), please clarify whether the p-value refers to a raw or adjusted p-value. Additionally, specify which enrichment analysis was conducted within the clusterProfiler package (Line 171).

Answer. We thank the reviewer for pointing this out. We would like to clarify that for the DEG analysis, we used the adjusted p-value, as indicated on line 163. Furthermore, we apologize for the error; we did not perform gene enrichment analysis using the clusterProfiler package in the present study. This information has now been removed from the Methods section

4. Clarify the timing of the functional assays (Lines 199–200). The current text does not explicitly define the "pre-stimulation," "simultaneous," and "post-stimulation" windows relative to the SARS-CoV-2 challenge. A diagram and clearer notation would help readers.

Answer. We thank the reviewer for their comments. A diagram illustrating the treatment schemes has now been included in Supplementary Figure 1G

Results

5. While IL-6 changes are presented, other cytokines did not show significant alterations in the U937 model. The authors should consider including a functional assay to support their conclusion of an IL-6-driven inflammatory response. This reviewer recognizes that conducting additional experiments at this stage may be challenging; however, an

experiment assessing the inflammatory potential of conditioned media from U937-infected cells would significantly strengthen the claims made in this manuscript.

Answer. We sincerely thank the reviewer for this thoughtful and constructive suggestion, and we greatly appreciate their understanding of the practical challenges associated with additional experiments at this stage. We have carefully considered their proposal to assess the inflammatory potential of conditioned media from U937-infected cells. However, after thorough consideration of the biological limitations of our experimental model, we respectfully believe that such an assay would not provide meaningful insights in this specific context. As the reviewer correctly noted, our U937-derived monocytes and macrophages did not produce significant levels of other pro-inflammatory cytokines, such as TNF-α and IL-1β, upon stimulation with either LPS or UV-inactivated SARS-CoV-2. This is a well-documented characteristic of this cell line, which exhibits a restricted inflammatory response profile compared to primary cells. Given that endothelial cell activation, the most common readout of conditioned media transfer experiments, is largely mediated by TNF-α and IL-1β, the absence of these cytokines in our conditioned media would likely yield negative results, regardless of IL-6 levels. Such an outcome would not meaningfully inform the IL-6-specific inflammatory potential we aim to interrogate, and could even be misinterpreted as a lack of biological relevance.

Thus, while we fully agree with the reviewer that assessing the functional consequences of SARS-CoV-2-induced inflammation is highly relevant, we propose that this important question is better addressed in future studies using primary human monocytes or macrophages, which possess a fully competent pro-inflammatory cytokine repertoire. We have now explicitly acknowledged this as a future perspective in the revised manuscript (lines 578-581), stating that conditioned media from primary SARS-CoV-2-infected mononuclear phagocytes should be tested on endothelial cells to fully capture the IL-6-driven inflammatory axis in the context of COVID-19.

6. While noted as a limitation (Line 519), the lack of VDR signaling target gene validation in the U937 model is a significant gap. The manuscript would be greatly strengthened if the authors could provide data for VDR signaling pathway to claim that impairment of this pathway drives the observed inflammatory response.

Answer. We thank the reviewer for the opportunity to clarify this point. In the new Supplementary Figure 1, we have included a transcriptomic characterization of the U937 macrophage model, confirming that these cells express all critical components of the VD3 biosynthesis and signaling pathway, with expression levels even higher than those observed in THP-1 cells. Furthermore, in Supplementary Figure S1F, we demonstrate that differentiation of U937 cells in the presence of calcitriol leads to increased expression levels of CD11b, a well-established VDR target gene. This finding confirms the functionality of the VDR pathway in U937 cells, which is consistent with previous reports

Figures

7. The legends for Figures 1–3 contain extensive repetition of dataset descriptions, patient group definitions, sample sizes, statistical procedures, and TPM quantification methodology. This information is re-stated in full in each legend despite being described in the Methods section (including the requested summary table). The authors should consolidate this shared information and replace the redundant text in each legend with a brief cross-reference (e.g., "Data from GSE216529; see Methods"). This would substantially improve readability without any loss of information.

Answer. We thank the reviewer for the suggestion. The changes have been implemented in the corresponding figure legends.

8. Figure 5 is an excellent diagram that effectively summarizes the findings of this manuscript. However, it would benefit from the removal of IL-1β and CXCL8, as these proteins were not found to be modulated in U937 cells.

Answer. We appreciate the reviewer's suggestion. However, although challenge with SARS-CoV-2 in our U937 cell model did not lead to significant production of IL-1β or CXCL-8, significant expression of these inflammatory factors was indeed observed in monocytes and/or mDCs from COVID-19 patients, as well as in MDMs infected with SARS-CoV-2 in vitro. We therefore consider these to be critical inflammatory factors in the SARS-CoV-2-induced cytokine storm within primary mononuclear phagocytes

Minor Comments and Technical Corrections

Abstract and Introduction:

9. The phrasing in lines 38–39 ("both COVID-19 patient monocytes and in vitro infected MDMs an NF-κB-dependent...") is difficult to follow and should be revised for better flow.

Answer. We thank the reviewer very much for the suggestion. The corresponding modification to the sentence has been made on lines 38-41 of the revised manuscript

10. Standardize the use of italics for all gene names, including CYP2R1, CYP27A1 (Line 107), CYP27B1 (Line 108), and CAMP (Line 113).

Answer. We thank the reviewer very much for this suggestion. We have now systematically applied italics to all gene names mentioned throughout this study. However, the examples pointed out by the reviewer on lines 107, 108, and 113 (CYP2R1, CYP27A1, CYP27B1, and CAMP) refer to the specific proteins rather than the genes that encode them; therefore, they have not been italicized.

11. Capitalize and normalize "Zika" (Line 120) and ensure the term "primary human mononuclear phagocytes" is used consistently (Line 124).

Answer. We have made the requested change

Results:

12. Please ensure strict adherence to official gene symbols throughout the section to maintain consistency, including in figures:

Use ITGAM for CD11b and ITGAX for CD11c (Lines 228–229).

Use DDX58 for RIG-I (Line 247).

Use NFKB1 for NF-κB1 (Line 251) and IL1B for IL-1β (Line 255).

Standardize symbols by removing special characters (e.g., use RXRA instead of RXRα and IL6 instead of IL-6 mRNA).

Correct "it" to "its" (Line 356) and replace "Moreover" with "Furthermore" (Line 378) for better transition.

Answer. We thank the reviewer very much for the suggestions. The modifications have been incorporated into both the text and the figures. For reasons of recognition within the academic community and to ensure greater clarity in the presentation of the results, some genes are referenced by their official name / the most widely recognized name.

Discussion:

13. Reduce the emphasis on the PRR/NF-κB activation section (Lines 428–447), as this information is heavily detailed in the Results. Instead, expand the section (Lines 465–483) discussing which aspects of the U937 cell line recapitulate primary human monocyte/macrophage responses, and integrate this more thoroughly with the Vitamin D results.

Answer. We thank the reviewer very much for the suggestions. We have shortened the paragraph in the following sections: lines 523-525 and 531-533. Additionally, we have expanded the section suggested by the reviewer, which now spans lines 506-569 in the revised manuscript

Figures

14. The legends for Figures 4 and 5 the term "kinetics" used in both legends is misleading. The data are presented as discrete box plots at two independent time points (4 and 24 h.p.s.), not as a continuous time-course. The authors should replace "Kinetics are shown for" with a more accurate phrase such as "Cytokine concentrations at 4 and 24 h.p.s. are shown for"

Answer. We have made the requested change

15. Line 641 contains a typographical error: "collected at 4 and h.p.s" is missing the second time point and should read "collected at 4 and 24 h.p.s."

Answer. We have made the requested change

Specific Questions for the Authors

16. The MDM data (GSE224845) involves infectious SARS-CoV-2 (Line 165), whereas the U937 validation uses UV-inactivated particles (Line 198). Did the authors observe any differences in the magnitude of VDR suppression when comparing infectious virus to inactivated particles?

Answer. We thank the reviewer for raising this important point. When we initiated our experiments in U937 cells, we had not yet obtained institutional approval for the use of infectious SARS-CoV-2; therefore, we were only permitted to use inactiva

Attachment Submitted filename: Response to Reviewers.pdf

# Peer review: Decision Letter 1

**DOI:** 10.1371/journal.pone.0357030.r003

**Authors:** Muhammad Iqhrammullah

11 Aug 2026

SARS-CoV-2 Infection Induces Functional Impairment of Vitamin D Receptor Signaling to Drive Interleukin 6-Dependent Hyperinflammation in Mononuclear Phagocytes.

PONE-D-26-15930R1

Dear Dr. Navas,

We’re pleased to inform you that your manuscript has been judged scientifically suitable for publication and will be formally accepted for publication once it meets all outstanding technical requirements.

Within one week, you’ll receive an e-mail detailing the required amendments. When these have been addressed, you’ll receive a formal acceptance letter and your manuscript will be scheduled for publication.

An invoice will be generated when your article is formally accepted. Please note, if your institution has a publishing partnership with PLOS and your article meets the relevant criteria, all or part of your publication costs will be covered. Please make sure your user information is up-to-date by logging into Editorial Manager at Editorial Manager® and clicking the ‘Update My Information' link at the top of the page. For questions related to billing, please contact billing support.

If your institution or institutions have a press office, please notify them about your upcoming paper to help maximize its impact. If they’ll be preparing press materials, please inform our press team as soon as possible -- no later than 48 hours after receiving the formal acceptance. Your manuscript will remain under strict press embargo until 2 pm Eastern Time on the date of publication. For more information, please contact onepress@plos.org.

Kind regards,

Muhammad Iqhrammullah, Ph.D

Academic Editor

PLOS One

Additional Editor Comments (optional):

Reviewers' comments:

Reviewer's Responses to Questions

**Comments to the Author**

Reviewer #1: All comments have been addressed

Reviewer #2: All comments have been addressed

**********

2. Is the manuscript technically sound, and do the data support the conclusions?

Reviewer #1: Yes

Reviewer #2: Yes

**********

3. Has the statistical analysis been performed appropriately and rigorously?

Reviewer #1: Yes

Reviewer #2: Yes

**********

4. Have the authors made all data underlying the findings in their manuscript fully available?

The PLOS Data policy

Reviewer #1: Yes

Reviewer #2: Yes

**********

5. Is the manuscript presented in an intelligible fashion and written in standard English?

Reviewer #1: Yes

Reviewer #2: Yes

**********

Reviewer #1: I have reviewed the revised version of the manuscript. The authors have adequately and satisfactorily addressed all the concerns I raised in the previous round of review. The manuscript has been substantially strengthened and is now suitable for publication. I recommend accepting this manuscript in its current form.

Reviewer #2: (No Response)

**********

what does this mean?). If published, this will include your full peer review and any attached files.

If you choose “no”, your identity will remain anonymous but your review may still be made public.

**Do you want your identity to be public for this peer review?** For information about this choice, including consent withdrawal, please see our Privacy Policy

Reviewer #1: No

Reviewer #2: No

**********

# Editor report: Acceptance letter

**DOI:** 10.1371/journal.pone.0357030.r004

**Authors:** Muhammad Iqhrammullah

PONE-D-26-15930R1

PLOS One

Dear Dr. Navas,

I'm pleased to inform you that your manuscript has been deemed suitable for publication in PLOS One. Congratulations! Your manuscript is now being handed over to our production team.

At this stage, our production department will prepare your paper for publication. This includes ensuring the following:

* All references, tables, and figures are properly cited

* All relevant supporting information is included in the manuscript submission,

* There are no issues that prevent the paper from being properly typeset

You will receive further instructions from the production team, including instructions on how to review your proof when it is ready. Please keep in mind that we are working through a large volume of accepted articles, so please give us a few days to review your paper and let you know the next and final steps.

Lastly, if your institution or institutions have a press office, please let them know about your upcoming paper now to help maximize its impact. If they'll be preparing press materials, please inform our press team within the next 48 hours. Your manuscript will remain under strict press embargo until 2 pm Eastern Time on the date of publication. For more information, please contact onepress@plos.org.

You will receive an invoice from PLOS for your publication fee after your manuscript has reached the completed accept phase. If you receive an email requesting payment before acceptance or for any other service, this may be a phishing scheme. Learn how to identify phishing emails and protect your accounts at https://explore.plos.org/phishing.

If we can help with anything else, please email us at customercare@plos.org.

Thank you for submitting your work to PLOS One and supporting open access.

Kind regards,

PLOS One Editorial Office Staff

on behalf of

Dr. Muhammad Iqhrammullah

Academic Editor

PLOS One
